# SEMG1/2 augment energy metabolism of tumor cells

**DOI:** 10.1038/s41419-020-03251-w

**Published:** 2020-12-11

**Authors:** Oleg Shuvalov, Alyona Kizenko, Alexey Petukhov, Olga Fedorova, Alexandra Daks, Andrew Bottrill, Anastasiya V. Snezhkina, Anna V. Kudryavtseva, Nikolai Barlev

**Affiliations:** 1 Institute of Cytology RAS, St-Petersburg, Russia; 2 Almazov National Medical Research Center, St-Petersburg, Russia; 3 University of Leicester, Leicester, UK; 4 Engelhardt Institute of Molecular Biology, Moscow, Russia; 5 MIPT, Dolgoprudny, Moscow Region, Moscow, Russia 141701; 6 IBMC Orekhovicha, Moscow, Russia 119435

**Keywords:** Cancer metabolism, Oncogenes

## Abstract

SEMG1 and SEMG2 genes belong to the family of cancer-testis antigens (CTAs), whose expression normally is restricted to male germ cells but is often restored in various malignancies. High levels of SEMG1 and SEMG2 expression are detected in prostate, renal, and lung cancer as well as hemoblastosis. However, the functional importance of both SEMGs proteins in human neoplasms is still largely unknown. In this study, by using a combination of the bioinformatics and various cellular and molecular assays, we have demonstrated that SEMG1 and SEMG2 are frequently expressed in lung cancer clinical samples and cancer cell lines of different origins and are negatively associated with the survival rate of cancer patients. Using the pull-down assay followed by LC-MS/MS mass-spectrometry, we have identified 119 proteins associated with SEMG1 and SEMG2. Among the SEMGs interacting proteins we noticed two critical glycolytic enzymes-pyruvate kinase M2 (PKM2) and lactate dehydrogenase A (LDHA). Importantly, we showed that SEMGs increased the protein level and activity of both PKM2 and LDHA. Further, both SEMGs increased the membrane mitochondrial potential (MMP), glycolysis, respiration, and ROS production in several cancer cell lines. Taken together, these data provide first evidence that SEMGs can up-regulate the energy metabolism of cancer cells, exemplifying their oncogenic features.

## Introduction

The family of cancer-testis antigens (CTAs) encompasses more than 200 tumor-associated antigens, which normally express in testis and placenta and function in reproduction. Upon malignization, CTA-coding genes become derepressed in transformed non-germ cells. Over-expression of CTAs is preferentially associated with poorly differentiated, metastatic tumors, which makes them a predictive marker of unfavorable survival prognosis for cancer patients^1,2^.

When de-repressed in non-germ cells, CTAs often serve as markers of specific immune activation since testis are immune-privileged organs and testis-specific proteins are immunogenic when they are expressed in tumors^3^. Therefore, a number of CTAs are considered as candidates for immune therapy^4,5^.

All CTAs can be divided into two groups—the ones located at X-chromosome (X-linked CTAs) and autosomal (non-X-linked) CTAs. X-linked CTAs (MAGE-A3, NY-ESO-1, etc) have been used in clinical trials as immune vaccines^6^ and hence they are generally better studied in the context of tumor biology.

Semenogelins 1 and 2 (SEMG1 and 2, respectively) are referred to non-X-linked (autosomal) CTAs and are the most abundant proteins of human semen^7^. SEMG1 is a 50 kDa protein, whereas SEMG2 has a molecular weight of 63 kDa. These proteins share 78% similarity and are composed of repetitive units^8^.

They are secreted to semen by seminal vesicles and then undergo rapid degradation by the prostate specific antigen (PSA, the kallikrein peptidase) to small peptides. In seminal fluid, SEMGs themselves and their proteolytic products perform a number of important functions. They regulate the motility^9^ and capacitation of sperm^10^, as well as provide it with the antibacterial defense^11^. Although SEMG1 and SEMG2 have a high percentage of homology, most of the information on the role of SEMGs in reproductive processes concerns SEMG1. Therefore, the functional role of SEMG2 remains virtually unexplored.

Not much is known about the mechanisms that control transcriptional re-expression of SEMGs in tumor cells^12^. SEMGs were found expressed in various malignancies including prostate^13^, lung^14^, and renal carcinoma^15^, as well as in some blood neoplasms^12^. The only known biological role of SEMGs in tumors described to date is related to prostate cancer in which SEMG1 served as a co-activator of androgen receptor^16^. Therefore, an important biological question emerges of whether the aberrant expression of CTAs in transformed cells is a consequence of gross deregulation of gene expression in tumors, or their expression provides additional advantages to cancer cells.

In the present work, we have shown that SEMG1 and SEMG2 are observed at different frequencies in various human cancer cell lines and are associated with poor prognosis for survival of patients. To uncover the molecular function(s) of SEMGs we have applied the proteomic approach to describe interactomes of SEMG1 and SEMG2. Functional analysis of SEMGs-associated proteins suggested their potential involvement in the regulation of metabolism. Using different cell models of human lung, breast, and pancreatic cancers we demonstrated that both SEMGs increased enzymatic activities of LDHA and PKM thereby up-regulating glycolysis, respiration, and superoxide production.

## Materials and methods

### Plasmids and cloning

Full-length CDS sequences of both human SEMG1 (NM_003007.4) and SEMG2 (NM_003008.2) were amplified by PCR from cDNA derived from MCF7 cell line with follows primers including restriction sites for subsequent cloning: *SEMG1_forward 5*′*-ATTGAATTCATGAAGCCCAACATCATCTTTGTAC-3*′, *SEMG1_reverse 5*′*-ATTCTCGAGTGTAAATAATGGGTTTCGGTCGTTG-3*′, *SEMG2_forward ACCGCGGCCGCTAGATGAAGTCCATCATCCTCTTTGTCC, SEMG2_reverse TTCTCGAGTGTAGATATTGGATTTCTGTCTTCATTATATTGTTG*. Amplified sequences were digested by *EcoR*I/*Xho*I (in the case of SEMG1) or *Not*I/*Xho*I (for SEMG2) and cloned to Pires-hr-1a vector in fusion with 3×-Flag tag. Then, sequences of 3×Flag-SEMG1 and 3×Flag-SEMG2 were cut by *EcoR*I/*Pml*I and *Not*I/*Pml*I and subcloned to LegoIG2 lentiviral vector (which allow selection by GFP fluorescence) purchased from Addgene and then were checked by sequencing.

To knockdown both SEMG1 and SEMG2, a lentiviral pGreenPuro vector bearing two different short hairpins against the corresponding genes (*sh_1, GCAAGTCTCAAAACCAGGTAACAATTCAT, sh_2 GAATGCCCTACATAAGACGACAAAATCAC*) or scramble sequence (CCTAAGGTTAAGTCGCCCTCG) were used.

Sequences encoding full-length LDHA (NM_005566.4) and PKM2 (NM_002654.6) were amplified using MCF7-derived cDNA with following primers: *PKM2_F_EcoRI ATTGAATTCACCATGTCGAAGCCCCATAGTGAAG, PKM2_R_NotI ATTGCGGCCGCCGGCACAGGAACAACACGC*. Amplified sequences were digested by *EcoR*I/*Xho*I (for LDHA) or *EcoR*I/*Not*I (for PKM2) and cloned to Pires-hr-1a vector in fusion with 3xFlag tag and checked by sequencing.

For GST pull-down experiments, pGEX-5X-1 vector for prokaryotic protein expression was used. Sequences encoding full-length SEMG1 (1-461 a.a.) and SEMG2 (1-562 a.a) were amplified using the previously describer primers for LegoIG2. Truncated SEMGs which lack exporting signaling peptides (SEMG1, 23-461a.a.; SEMG2, 23-562 a.a.) were amplified using following forward primers: *Semg1_F_trunc_EcoRI ATTGAATTCCAAAAAGGTGGATCAAAAGGCC, Semg2_F_trunc_XmaI ATTCCCGGGTCAAAAAGGTGGATCAAAAGGCC*. Reverse primers for both SEMGs were previously described. *Eco*R*I*/*Xho*I and *Eco*R*I*/*Xma*I digested PCR products were cloned into pGEX-5X-1 vector in fusion with sequence encoding glutathione-transferase (GST).

### Cell lines manipulations

All cell lines used in this study were purchased from ATCC: lung adenocarcinoma lines H1299, H520, and H1650; breast carcinoma MCF7 and MDA-MB-231, pancreatic adenocarcinoma Mia-Paca2 and ASPC1, cervix carcinoma Hela. All cells were propagated in RPMI medium (Gibco) supplemented with 10% fetal bovine serum (FBS) (Gibco) and were grown at 37 °C in a humidified atmosphere with 5% CO_2_.

#### Transient transfection

Transfections of H520, MCF7, and MDA-MB-468 cells were carried out using Lipofectamin 2000^®^ (Invitrogen, USA) according to the manufacturer’s protocol. All experiments (western blotting and flow cytometry) were performed three days after transfection. The efficiency of transfection was checked by western blotting.

#### Establishing cell lines with stable overexpression of SEMG1 or SEMG2

Lentiviral transduction of H1299, MDA-MB-231, and Mia-Paca 2 cells by LegoiG2 vector bearing SEMG1 or SEMG2 was carried out according to the protocol described previously. Transduction by native LegoIG2 vector was performed for control cells. A week later, transduced cells were subjected to GFP-based sorting by BD FACS ARIA III (USA). Efficiency of sorting was controlled by flow cytometry and was in a range of 85–90% for all cell lines.

#### Establishing cell lines with knockdown of SEMG1and SEMG2

Lentiviral transduction of H520 cells by pGreenPuro vector bearing sh1, sh2, or scramble was carried out according to the standard protocol.

### GST pull-down assay

Expression of GST-SEMG1 and GST-SEMG2 recombinant proteins was carried out in *E. coli BL21 Rosetta* strain. Native pGEX-5X-1 vector, encoding GST was used as control. Optimized expression of GST-SEMG1 and GST-SEMG2 was obtained using truncated SEMG1 and SEMG2, and 4 h induction with 0.4 mM IPTG at +37 °C. Recombinant proteins were purified using glutathione sepharose 4B beads (GE Life Sciences, USA).

To minimize unspecific binding, 50 µg of GST was used for pre-incubation with MCF7 cell extract during 3 h. Than 50 µg of GST, GST-SEMG1, and GST-SEMG2 were used for pull-down during 3 h followed by washing steps. Interacting proteins associated with the recombinant proteins were eluted by adding Laemmli buffer and resolved by SDS-PAGE.

### LC-MS/MS protein identification

Protein samples were excised from the gel before trypsinolysis and then were subjected to liquid chromatography on the reversed-phase column (Dionex, UK) followed by elution at reversed-phase column Waters Symmetry C18 100Е (Waters, UK). The analysis of protein fractions was carried out at 4000 Q-Trap (Applied Biosystems, UK) mass-spectrometer. Ion spectra were analyzed by using MASCOT49 software and UniProtKB/Swiss-Prot50 databases. The considered proteins were only those for which at least two different peptides with *p* < 0.05 were obtained. Mass-spectrometry data are deposited at the Mendeley Data as “Proteins associated with SEMG1 and SEMG2”, v1, 10.17632/24nng467bp.1.

### Western blotting

For western-blotting following antibodies were used: anti-SEMG1 (1:1000, PA5-30168, Invitrogen), anti-SEMG2 (1:1000, PA5-42099, Invitrogen), anti-Flag (1:1000, M2, Sigma, USA), anti-LDHA (1:5000, #3582, CST), anti-PKM2 (1:1000, PA5-28623, Invitrogen), and anti-β-actin (1:1000, A-2228, Sigma, USA). The secondary antibodies were anti-mouse and anti-rabbit (1:10,000; Sigma, USA).

### Co-immunoprecipitation

HEK293T cells were transfected 48 h before co-immunoprecipitation assay with Pires-hr-1a encoding 3 × Flag-SEMG1 and 3 × Flag-SEMG2. Control cells were transfected with native vector. Cells were lysed in the hypotonic buffer (10 mM Tris-HCl, pH 7.4, 10 mM NaCl, 10 mM EDTA, 1 mM NaF 0.25%, Triton X-100 and protease inhibitor cocktail) on ice during 10 minutes. Next, 150 mM NaCl was added to increase the ionic strength of the buffer (50 mM Tris-HCl, pH 7.4, 150 mM NaCl, 20 mM EDTA, 0,1%, Triton X-100 and protease inhibitor cocktail) with additional 10 min of lysis on ice. Cell lysates were cleared by centrifugation and were then incubated with anti-Flag M2 (Sigma, USA) agarose beads for 4 h followed by three washing steps with TBS buffer. Recovered immune complexes were eluted by boiling in Laemmli buffer and were subjected to western blot analysis.

For reciprocal co-immunoprecipitation, H520 cells expressing both SEMGs were used. These cells were transiently transfected by pCDH vector encoding 3 × Flag-tagged LDHA, PKM2 or empty vehicle. Lipofectamine 2000 was used as transfecting reagent (according to manufacturer’s recommendation). Co-immunoprecipitation of endogenous proteins was carried out 48 h post-transfection using anti-Flag M2 (Sigma, USA) agarose beads as was described for HEK293T cells.

### RNA isolation and relative quantification RT-PCR

Total RNA was extracted from the cultured cells using TRIzol Reagent (Thermo Fischer Scientific, USA) according to the manufacturer’s instructions. For cDNA synthesis the RevertAid First Strand cDNA Synthesis Kit (Evrogene) was used.

Quantitative Real Time PCR was performed using SsoFast EvaGreen Master Mix (BioRad, CA, USA) and BioRad CFX-96 real time system (BioRad, CA, USA). The ΔΔCt method was used to calculate relative expression. Sample’s Ct values were normalized to GAPDH. The oligonucleotides used for qPCR were as follows: GAPDH sense 5′-GAGGTCAATGAAGGGGTCAT-3′ and antisense 5′-AGTCAACGGATTTGGTCGTA-3′; PKM2 5′-TTGCTTCCCCAGTCTGAGTC-3′, 5′-ACTTCTCTTTGTTTTGGGCG-3′; LDHA 5′-GGAGATCCATCATCTCTCCC-3′, 5′-GGCCTGTGCCATCAGTATCT-3′. Amplifications were performed in triplicates.

### Enzymatic activity assay

To assess LDHA and PKM2 activity, LDH activity kit (Sigma), and PKM activity kit (Sigma) were used, respectively. One hundred thousands of cells were planted a day before experiment in triplicates. Measurements were performed according to manufacturer’s protocol.

### Mitochondria membrane potential (MMP) assessment

To visualize MMP, MitoTracker Red CMXRos fluorescent dye was applied. Cells were incubated with 250 nM of MitoTracker dye followed by either flow cytometry quantification by Guava EasyCyte 8 (EMD Millipore, USA) or PFA fixation with subsequent DAPI-contained mounting and confocal microscopy analysis by fluorescent microscope with 540–550 nm excitation filter plus >575 nm (long pass) filter tandem. Values of median were used for quantification in flow cytometry experiments.

### SeaHorse profiling

For directly measuring of the oxygen consumption rate (OCR) and Extracellular acidification rate (ECAR) of cells, Seahorse XFe24 Analyzer (Agilent, USA) and Mito Stress kit (Agilent, USA) were used. Thirty-thousand of cells were planted on Seahorse 24-well plate in five replicates. On the next day, the measurements of ECAR and OCR were performed according to manufacturer’s protocol. Resulting data were analyzed by Wave software (Agilent, USA).

### Measurement of ROS production

The production of endogenous superoxide was quantified by using Muse ROS assay (dihydroethidium-based) kit (EMD Millipore, USA) in accordance with manufacture’s protocol followed by flow cytometry at Guava easy cite 8 (EMD Millipore, USA) or CytoFLEX (Beckman Coulter, USA). Values of median were used for quantification.

### Bioinformatics analysis

The expression levels of SEMG1 and SEMG2 in clinical samples of lung cancer patients and in the corresponding normal tissue were analyzed by Phantasus software (https://artyomovlab.wustl.edu/phantasus/). Pre-calculated expression values were log-transformed and quantile normalized using the R (v3.5.2) statistical language in the R studio software (v1.1.456). Heatmaps were produced using the ggplot2 library. Microarray expression datasets were obtained from the open-source database GEO Datasets (GSE36471—lung adenocarcinoma samples, GSE3268-paired squamous cell lung cancer and normal tissue samples).

Kaplan–Meier plots calculating the correlations between SEMG1 or SEMG2 expression levels and survival outcomes of cancer patients were obtained using the PPIsurv software (http://www.bioprofiling.de/GEO/PPISURV/ppisurv.html). The microarray expression data were obtained from the open-source database GEO Datasets (GSE31192—pregnancy-associated breast cancer, GSE116959—lung adenocarcinoma). Expression levels of the SEMG1 and SEMG2 genes in tumors and peritumoral normal tissues have been compared.

### Statistical analysis

All data are demonstrated as mean ± standard deviation (SD) of at least three biological replicates. The statistical tests were performed using Graphpad Prism (Version 7.04) software. Statistical significance was analyzed using Student *t*-test, *P* < 0.05 was considered significant and is denoted as *, *P* < 0.01 as **.

## Results

### SEMG1 and SEMG2 are frequently over-expressed in human cancer cell models and clinical samples of lung cancer

**Fig. 1:**
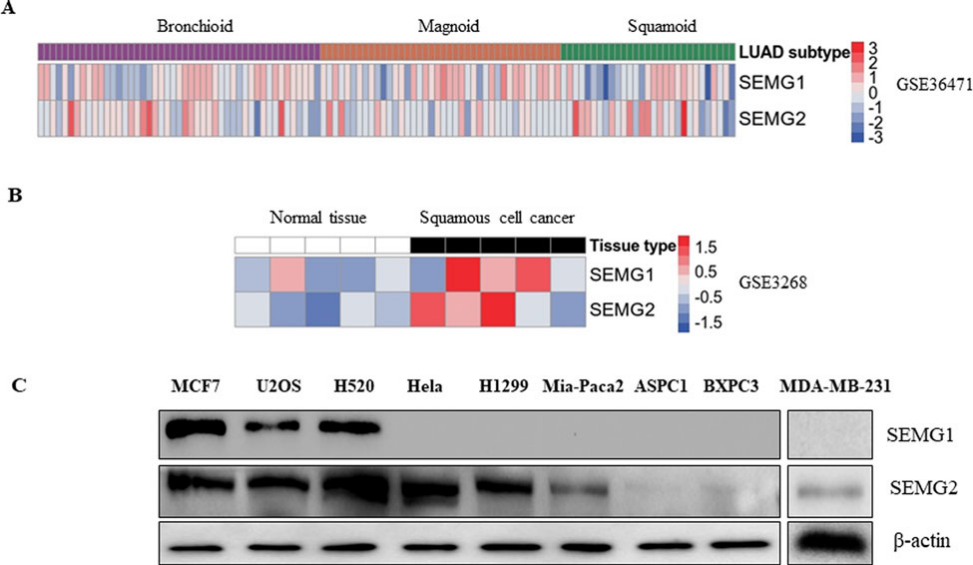
SEMG1 and SEMG2 are frequently expressed in human cancer cell lines of different origin and clinical samples. **A** Heat map of SEMG1 and SEMG2 expression in human lung cancer cell models (GSE36471) based on the RNA-seq data. Pre-calculated expression values (Phantasus software) were log-transformed and quantile normalized using the R (v3.61) statistical language in the R studio software (v1.2.5001). Heatmaps were produced using the ggplot2 library. **B** Heat map of SEMG1 and SEMG2 expression in clinical samples of lung squamous carcinoma (GSE3268) and the corresponding normal tissue. **C** Western-blot analysis of several human tumor cell lines of different origin for SEMG1 and SEMG2 expression.

Because SEMGs were found present in lung cancer^14^, we first decided to compare the mRNA levels of SEMGs in the panel of human lung cancer cell lines (small cell carcinomas, squamous cell carcinomas and adenocarcinomas). To this end, we employed the bioinformatics in silico approach using Phantasus analyzer (https://genome.ifmo.ru/phantasus) to inspect the Cancer Genome Atlas (TCGA) data. As shown in Fig. 1A, both SEMGs are expressed in all types of lung cancer cell lines tested but the mRNA level of SEMG1 in general was higher than that of SEMG2.

Using the same software, we have also analyzed the expression of SEMGs in clinical samples of squamous carcinoma and the corresponding healthy tissue. Figure 1B demonstrates that the expression level of SEMGs, especially SEMG1 was significantly higher in carcinoma samples versus normal tissues.

In addition, we have analyzed SEMGs expression in pregnancy-associated breast cancer and lung adenocarcinoma datasets obtained from the open-source GEO database. As shown in Fig. 1S (Supplement Figures), the mRNA levels of SEMG1 and SEMG2 in tumor samples were higher than in peritumoral tissues.

**Fig. 2:**
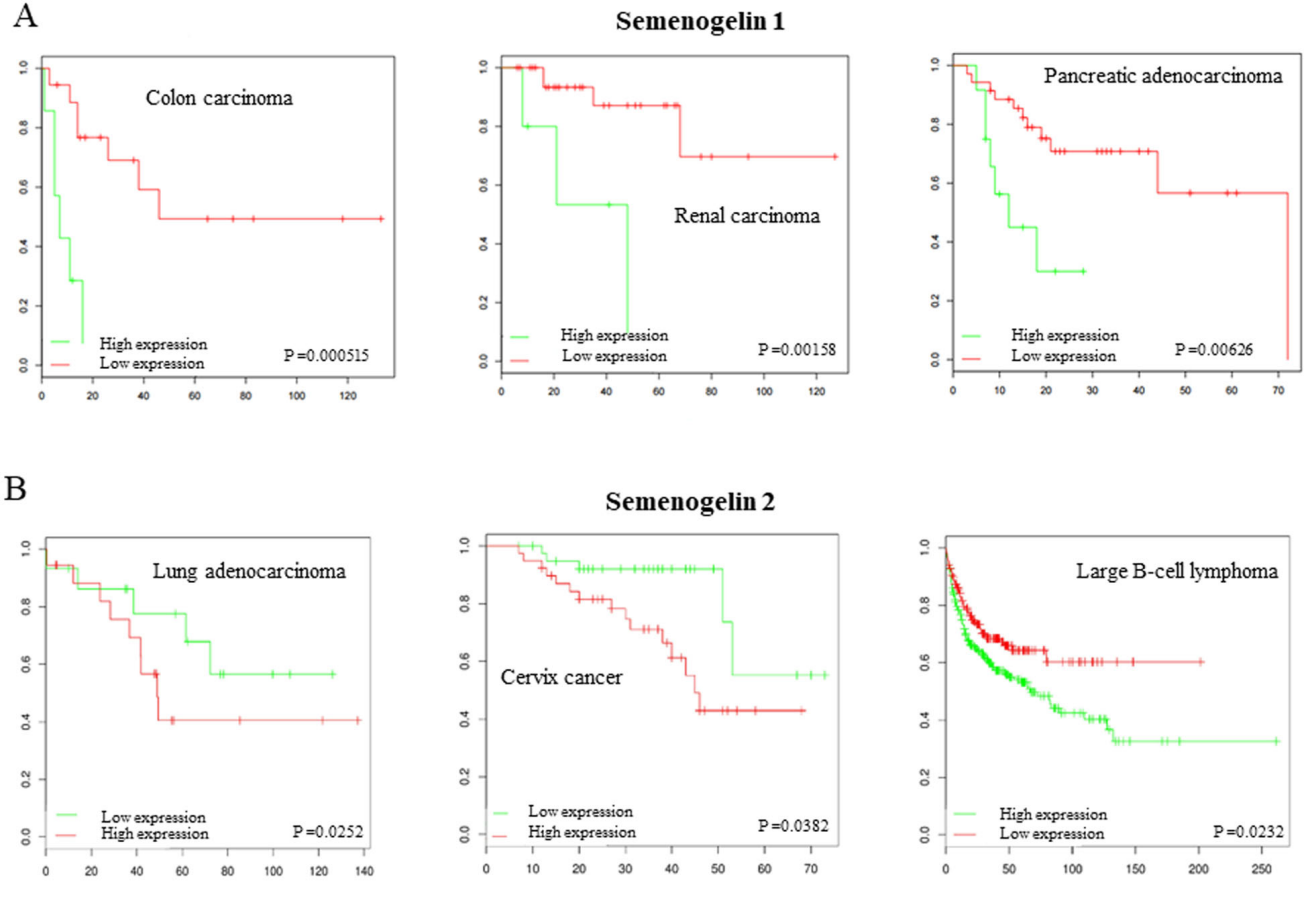
The expression level of SEMG1and SEMG2 negatively associated with survival rate of cancer patients. Kaplan–Meier plots demonstrating the associating between mRNA levels of **A** SEMG1 or **B** SEMG2 and outcome of patients with different types of cancer. PPIsurv software (http://www.bioprofiling.de/GEO/PPISURV/ppisurv.html) using algorythms described by Antonov et al.^17^ and publically available Gene Expression Omnibus (GEO) microarray data were applicated.

Furthermore, we have also analyzed the panel of human cancer cell lines of different origin by western blotting. In contrast with the bioinformatics data, Fig. 1C shows that SEMG2 was expressed at the protein level in almost all cell lines tested excluding two lines of pancreatic cancer whereas SEMG1 was detected in only three cell lines—MCF7 (breast carcinoma), U2OS (osteosarcoma) and H520 (NSCLC). In addition, we also included lung cancer cell line (H520) and two non-transformed human lung fibroblast cell lines—DF2 and WI-38 (Fig. 2S). SEMG1 and SEMG2 were expressed in H520 adenocarcinoma cell lines but not in normal human fibroblasts.

Taken together, these data show that SEMG1 and SEMG2 are frequently expressed in various human malignancies at both mRNA and protein levels.

### The mRNA level of SEMG1 and SEMG2 in different malignancies is predominantly negatively associated with patient’s outcome

To study if the expression of SEMGs in malignancies is associated with patient’s survival, we have used the PPIsurv software^17^. For lung cancer patients, the same dataset (GSE36471), which was used for calculation of SEMGs expression (Fig. 1A) was analyzed. These data are presented as Kaplan–Meier plots (Fig. 2A, B) and are summarized in Tables 1 and 2.

**Table 1:** The expression level of SEMG1 is negatively associated with survival rate of cancer patients.

Dataset (GEO ID)	Type of cancer	Effect on survival	*P*-value
METABRIC	Breast carcinoma	Positive	0.000621
TCGA_PAAD	Pancreatic adenocarcinoma	Negative	0.0521
TCGA_COAD	Colon adenocarcinoma	Negative	0.0382
GSE36471	Lung adenocarcinoma	Negative	0.0252
GSE10846	Large B-cell lymphoma	Negative	0.0232

**Table 2:** The expression level of SEMG2 is negatively associated with survival rate of cancer patients.

Dataset (GEO ID)	Type of cancer	Effect on survival	*P*-value
GSE7390	Breast carcinoma	Positive	0.0355
TCGA_COAD	Colon adenocarcinoma	Negative	0.000515
TCGA_KIRP	Renal cell papillary carcinoma	Negative	0.00158
GSE10846	Large B-cell lymphoma	Negative	0.00337
TCGA_PAAD	Pancreatic adenocarcinoma	Negative	0.00626
TCGA_SARC	Sarcoma	Negative	0.00707
TCGA_UCS	Uterus carcinoma	Negative	0.0292
TCGA_BRCA	Invasive breast carcinoma	Negative	0.0183

These data demonstrated that in the majority of datasets analyzed the mRNA level of SEMG1 and SEMG2 was negatively associated with survival rates of patients. This suggests that the expression of SEMGs favors tumor development.

### SEMG1 and SEMG2 interact with different proteins

**Fig. 3:**
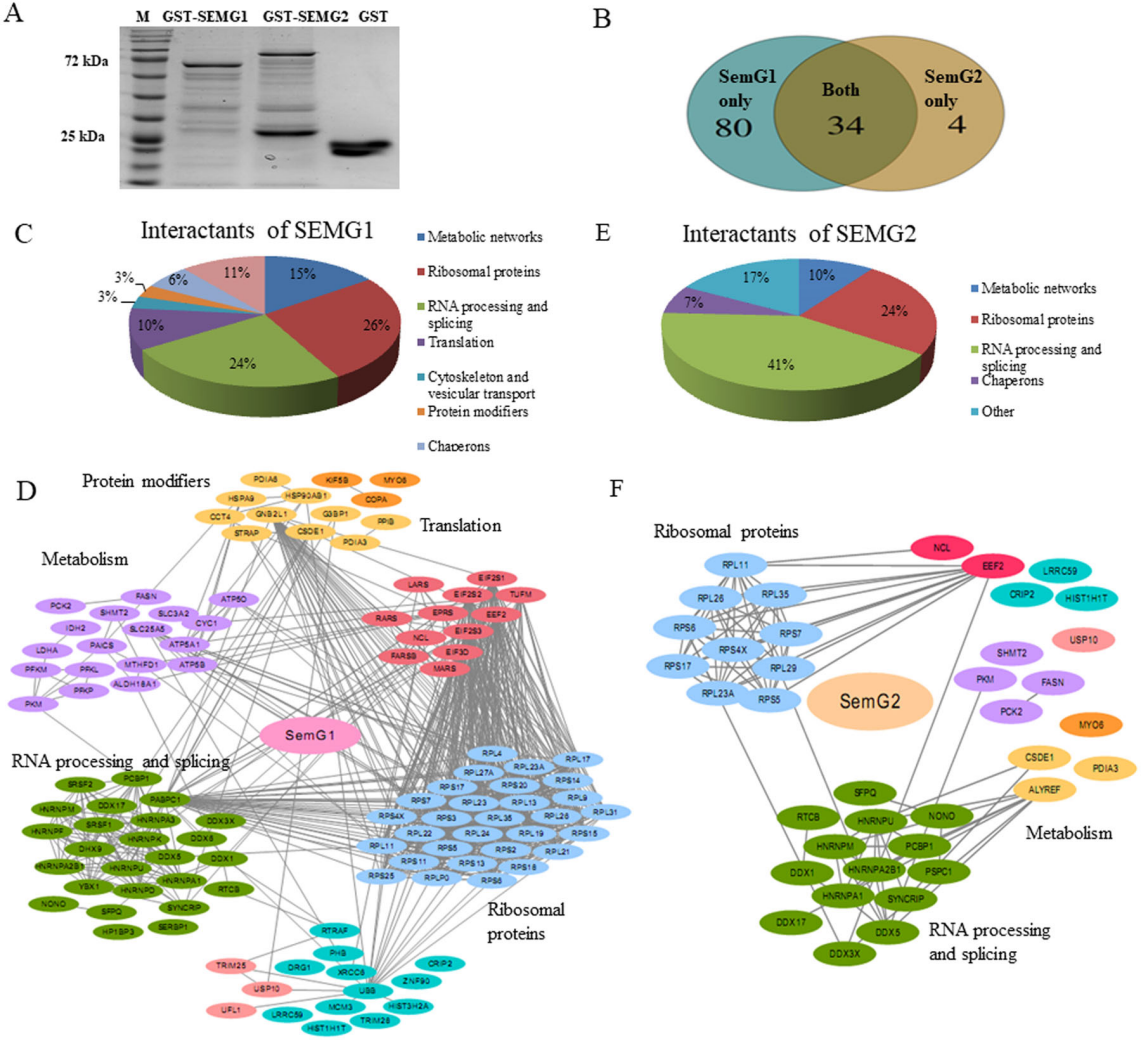
Identification of proteins associated with SEMG1 and SEMG2. **A** Coomasie staining of PAAG after separation of proteins interacted with recombinant SEMG1 and SEMG2. **B** Overlap of proteins associated with SEMG1 and SEMG2. Distribution by function of proteins associated with SEMG1 (**C**, **D**) and SEMG2 (**E**, **F**).

To elucidate the potential function of SEMGs in malignant cells, we decided to carry out GST pull-down assay to identify the SEMGs-interacting proteins. First, we optimized the expression of full-length recombinant SEMG1 (1-461 a.a.) and SEMG2 (1-562 a.a.) fused with GST in several bacterial expressing strains. However, the high degradation rate of both SEMGs was obtained (data not shown). To overcome this complexity, we have constructed vectors carrying recombinant SEMGs lacking signal peptides—SEMG1 (23-461 a.a.) and SEMG2 (23-562a.a.) fused with GST. Equivalent amounts of purified GST-SEMG1, GST-SEMG2, and GST (control) proteins were incubated with whole cell extract of human breast carcinoma MCF7 cells, separated in PAAG (Fig. 3A) and then subject to LC-MS/MS with subsequent identification of associated proteins. Mass-spectrometry data are deposited at Mendeley Data 10.17632/24nng467bp.1.

We have identified 119 proteins associated with either SEMG1 or SEMG2 (Supplementary, Lists 1, 2, 3, 4, 5, and 6). Interestingly, among these, only 38 proteins were identified as interactors of both SEMG1 and SEMG2, whereas the majority of proteins was associated only with SEMG1 and only four proteins were unique for SEMG2 (Fig. 3B and Supplementary, Lists 4 and 5). The functional annotation of interacting proteins identified has demonstrated the potential difference between SEMG1 and SEMG2 functions (Fig. 3B–F). The proteins associated with SEMG1 only (Supplementary Fig. 3_Suppl, List 4) were enriched with ribosomal proteins (27%), metabolic enzymes and transporters (18%), RNA processing and splicing factors (15%), regulators of translation (13%), signaling proteins (4%), chaperons (7%), other protein modifiers (4%), and other proteins (12%). At the same time, proteins associated with both SEMG1 and SEMG2 (Supplementary, List 6) displayed different distribution ratio between the functional groups: RNA processing and splicing (45%), components of ribosomes (24%), metabolic enzymes and transporters (11%), chaperons (5%), and proteins with other functions (15%).

So, in comparison with SEMG1, both quantitative and functional diversity of SEMG2 interacting proteins is significantly decreased and majority of its interactants (41%) take part in RNA processing and splicing.

### SEMG1 and SEMG2 physically interact with metabolic enzymes and enhance their activity

Among all functional groups of proteins associated with SEMGs, we have focused on key enzymes of cancer-related metabolism. Indeed, LDHA, PKM, and IDH2 are well-known players in the upregulation of cancer metabolism which are strongly associated with aggressive and invasive behavior of tumors and shortened patient’s survival^18,19^.

**Fig. 4:**
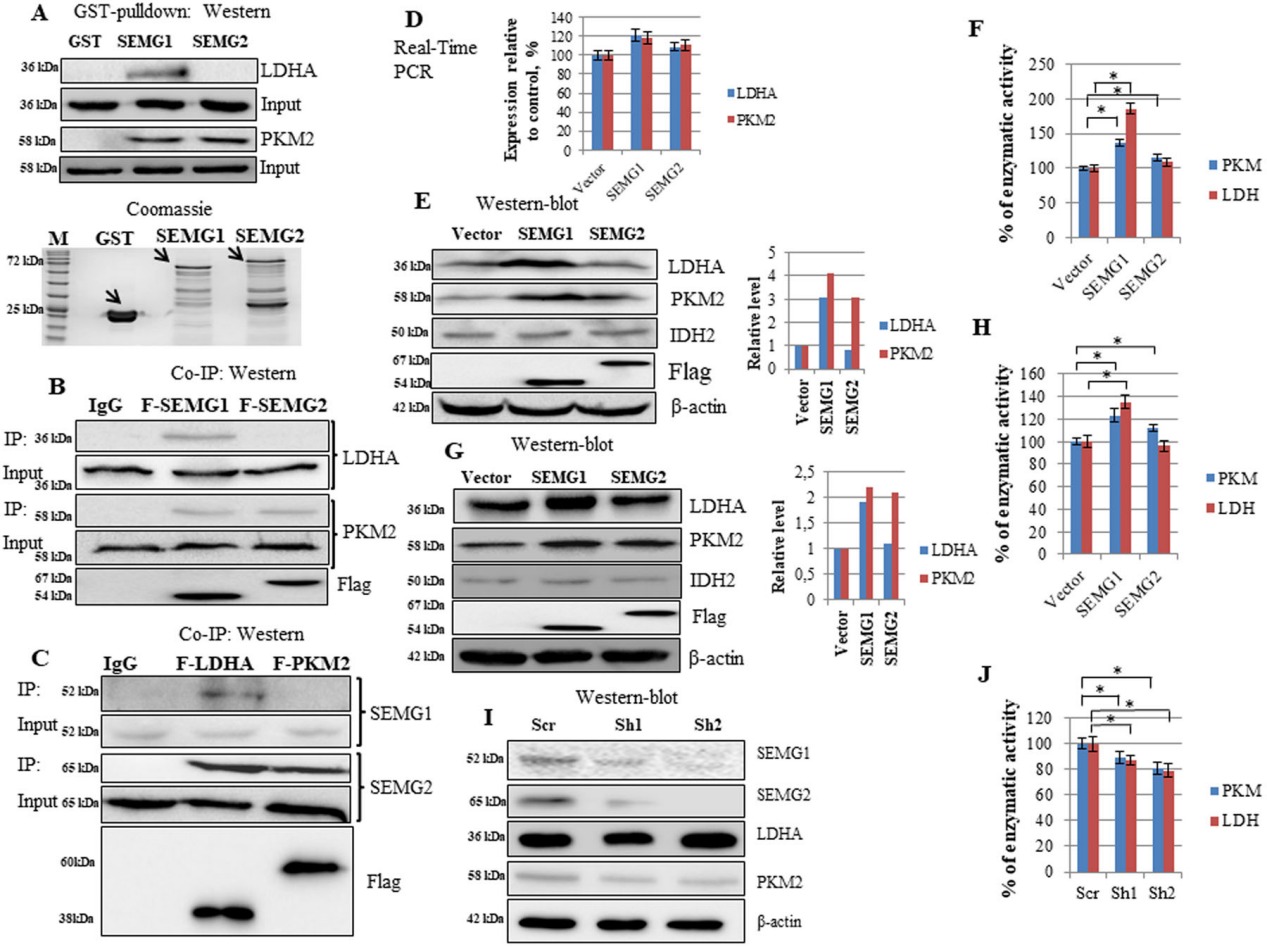
SEMG1 and SEMG2 interact with PKM2 and LDHA, upregulates their protein level and enzymatic activity. Recombinant SEMG1 and SEMG2 bind PKM2, whereas SEMG1 only binds LDHA in GST pull-down assay followed by western-blotting. **B** 3×Flag-tagged SEMG1 and SEMG2 bind PKM2, whereas SEMG1 only binds LDHA in co-immunoprecipitation. **C** 3×Flag-tagged PKM2 interacts with both endogenous SEMG1 and SEMG2 in H520 cells, whereas 3xFlag-tagged LDHA binds SEMG1 only (co-immunoprecipitation). **D** Overexpression of SEMG1 and SEMG2 in H1299 cell line does not alters the mRNA levels of PKM2 and LDHA (Real-Time PCR). The stable overexpression of SEMG1 and SEMG2 increase the protein level and enzymatic activity of PKM2, whereas the overexpression of SEMG1 only elevates the protein level and enzymatic activity of LDHA in **E**, **F** H1299 cells and **G**, **H** Mia-Paca 2 cells. Knockdown of SEMG1 and SEMG2 in H520 cells decreases the protein level (**I**) and enzymatic activity (**J**) of PKM2 and LDHA. Three biological replicates were used for all quantifications, data are presented as mean ± S.D., *P < 0.05.

As a first step, we have verified results of mass-spectrometry. To do this, we repeated the GST pull-down experiment with recombinant SEMG1 and SEMG2 and probed the precipitated material with antibodies against LDHA, PKM2 and IDH2 using western blotting (Fig. 4A). We have also confirmed this result by co-immunoprecipitation of Flag-tagged SEMG1 and SEMG2 followed by western blotting with the same antibodies (Fig. 4B) as well as by reciprocal co-immunoprecipitation of Flag-tagged LDHA and PKM2 with endogenous SEMG1 and SEMG2 in H520 cells (Fig. 4C). Interestingly, PKM2 and IDH2 interacted with both SEMGs, whereas LDHA bound SEMG1 only.

To investigate the influence of SEMGs on metabolic enzymes and energy metabolism in several cancer models, we have established H1299 (non-small lung adenocarcinoma), MDA-MB-231 (breast carcinoma) and Mia-Paca2 (pancreatic adenocarcinoma) cell lines with stable overexpression of 3×Flag-tagged SEMG1 or SEMG2. The respective cell lines expressing the corresponding empty vector were used as control. Using H1299 cells, we demonstrated by Real-Time PCR that overexpression of SEMG or SEMG2 did not significantly alter mRNA levels of PKM2 and LDHA (Fig. 4D).

However, overexpression of SEMG1 and SEMG2 in H1299 and Mia-Paca2 cells increased the protein level and enzymatic activity of PKM2, whereas the overexpression of SEMG1 alone elevated the protein level and enzymatic activity of LDHA (Fig. 4E–H).

At the same time, knockdown of both SEMGs by sh_RNA_1 led to the decrease of protein level and enzymatic activity of PKM2 and LDHA (Fig. 4I, J). Sh_RNA_2 decreased the protein level of PKM2 only. However, sh_RNA_2 inhibited the enzymatic activity of both PKM2 and LDHA (Fig. 4J).

Taken together, we concluded that SEMG1 and SEMG2 differently interacted with glycolytic enzymes PKM2 and LDHA, increasing their protein levels and enzymatic activities.

### SEMG1 and SEMG2 upregulates mitochondrial membrane potential, glycolysis, and respiration

Since both SEMG1 and SEMG2 affected the activity of crucial metabolic enzymes of glycolysis, we next decided to assess whether these proteins can regulate the cancer-related metabolism because glycolysis is a well-known hallmark of cancer.

**Fig. 5:**
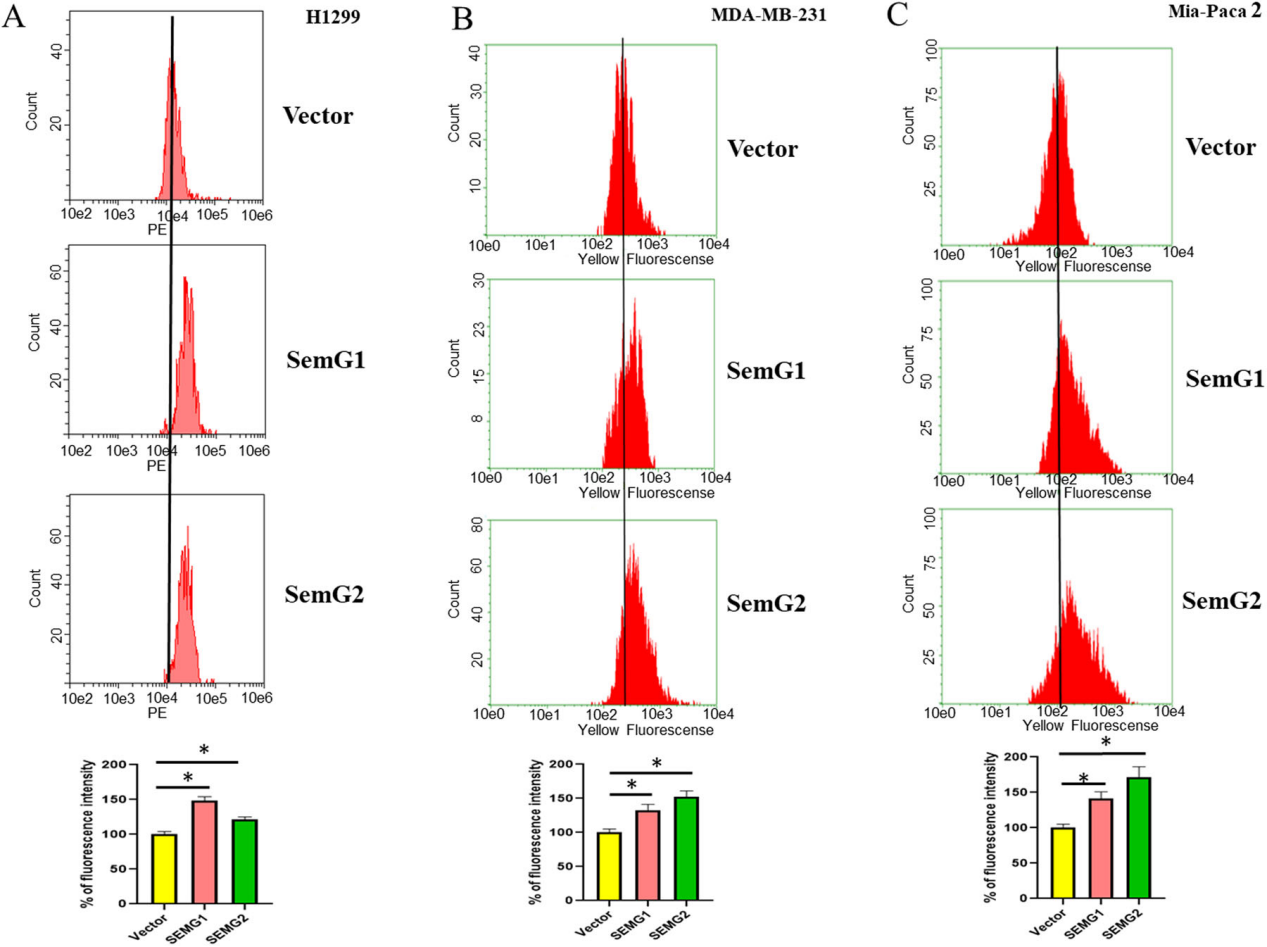
SEMG1 and SEMG2 increase mitochondrial membrane potential (MMP). The flow cytometry of MitoTracker fluorescence in **A** H1299, **B** MDA-MB-231 and **C** Mia-Paca 2 cells with stable overexpression of SEMG1, SEMG2 or corresponding vehicle. Quantification was done in triplicates, data are presented as mean ± S.D., *P < 0.05.

**Fig. 6:**
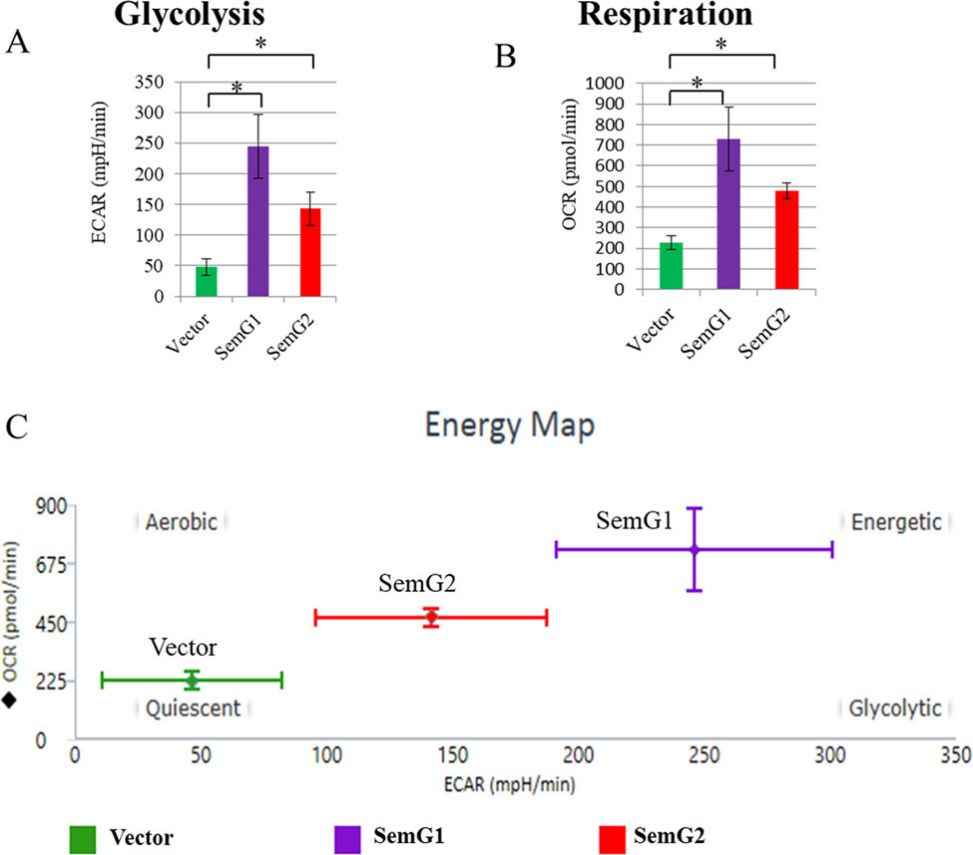
SEMG1 and SEMG2 up-regulate glycolysis and respiration. SeaHorse® profiling of H1299 cells with stable overexpression of SEMG1, SEMG2 or control vector. SEMGs elevate **A** glycolysis and **B** respiration; ECAR extracellular acidification rate, OCR oxygen consumption rate. **C** Energy map showing increased energetic status of SEMGs-overexpressing related to control cells. Three biological replicates were used for quantification, data are presented as mean ± S.D., *P < 0.05.

To this end, we used H1299, MDA-MB-231, and Mia-Paca2 cells with SEMG1 and SEMG2 overexpression to test if SEMGs affect mitochondrial membrane potential (MMP). MMP reflects the energy status of cells. The value of MMPs’ hyperpolarization positively correlates with the invasiveness and aggression of tumor^20^. We incubated cells with MitoTracker CMXROS, which is sensitive to MMP followed by flow cytometry to quantify its fluorescence. Results shown on Fig. 5A–C demonstrate that both SEMGs significantly increased MMP (up to 154% over respective control cells) of all cell lines tested. To extend our observations, we examined for MMP two additional breast cancer cell lines, MDA-MB-468 (Fig. 4A_Suppl) and MCF7 (Fig. 5A_Suppl), transiently transfected with SEMG1 or SEMG2 vectors. A corresponding empty vector was used as control. All transfected cell lines were subsequently analyzed by flow cytometry using MitoTracker. Results shown in Fig. 4B, D_Suppl and Fig. 5B, D_Suppl demonstrate that ectopically expressed SEMG1 and SEMG2 up-regulate MMP in all cell lines. We also stained the control, SEMGs overexpressing H1299, and Mia-Paca2 cell lines with MitoTracker followed by confocal microscopy (Fig. 6_Suppl).

**Fig. 7:**
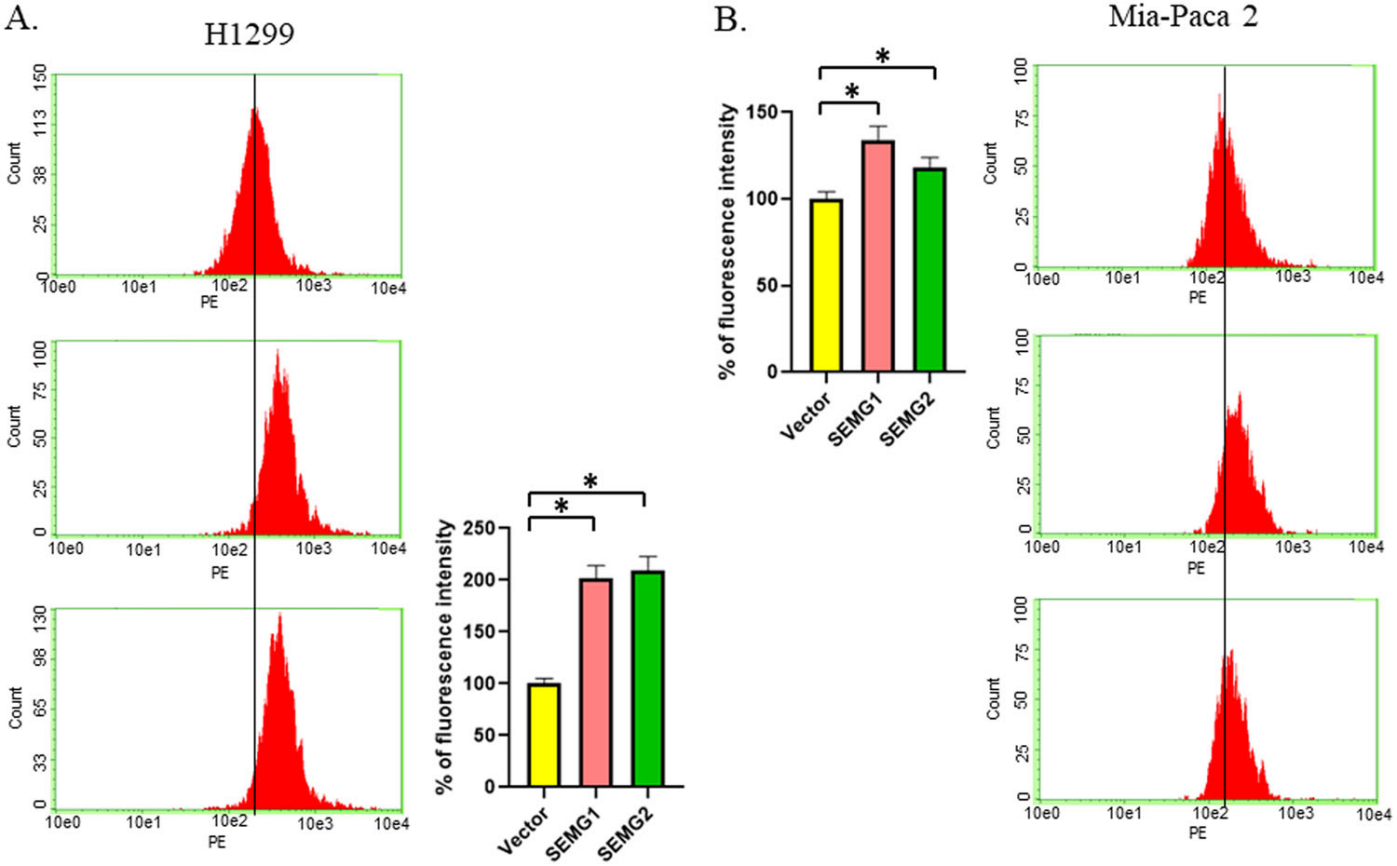
SEMG1 and SEMG2 enhance ROS production. The flow cytometry of DHE (which predominantly detects superoxide) fluorescence in **A** H1299 or **B** Mia-Paca 2 cells overexpressing SEMG1, SEMG2 or control vehicle. Fluorescence Data of three experiments were used and are presented as mean ± S.D., *P < 0.05.

In contrast, knockdown of SEMG1 and SEMG2 (Fig. 7A_Suppl) led to the decrease of MMP. Taken together, these results suggest that both SEMG1 and SEMG2 enhance the fluorescence intensity of MitoTracker, hence reflecting an increase of MMP and possible cancer aggressiveness.

To assess in details the influence of SEMGs on energy metabolism of cancer cells we have carried out the SeaHorse profiling of Mia-Paca2 cell line overexpressing SEMG1, SEMG2, or control vector. Our results suggest that overexpression of SEMG1 increased rates of glycolysis (ECAR) and respiration (OCR) approximately 5 and 3 times, respectively (Fig. 6A, B). At the same time, overexpression of SEMG2 up-regulated glycolysis and respiration approximately 3.5 and 2.5 times, respectively (Fig. 6A, B). So, the expression of both SEMGs made cells more energetic (Fig. 6C).

Taken together, these data clearly demonstrate that SEMG1 and SEMG2 enhance energy metabolism of cancer cells.

### SEMG1 and SEMG2 upregulate ROS production

In light of the results showing SEMGs-mediated upregulation of glycolysis and respiration, we aimed to test whether SEMGs can influence on ROS production because alterations in energy metabolism is usually linked to oxidative stress^21^. We used H1299 and Mia-Paca2 cells with stable overexpression of SEMGs and control cells to assess the production of superoxide, the main source of the respiratory chain. Moreover, we also examined MDA-MB-468 and MCF7 breast cancer cell lines transiently transfected with either SEMG1, SEMG2, or an empty vector as control for the ROS production.

Results shown in Fig. 4C, E_Suppl, 5C, E_Suppl, and Fig. 7A and B demonstrate that both SEMGs significantly increased ROS production in all cell lines. In contrast, knockdowns of SEMG1 and SEMG2 (Fig. 7C, E_Suppl) led to the opposite results, i.e., caused a decrease of DHE fluorescence manifesting an attenuation of ROS.

These observations are in accordance with the hypothesis of SEMGs-mediated upregulation of glycolysis and respiration.

### The overexpression of PKM2 and LDHA in H1299 cells increase MMP

**Fig. 8:**
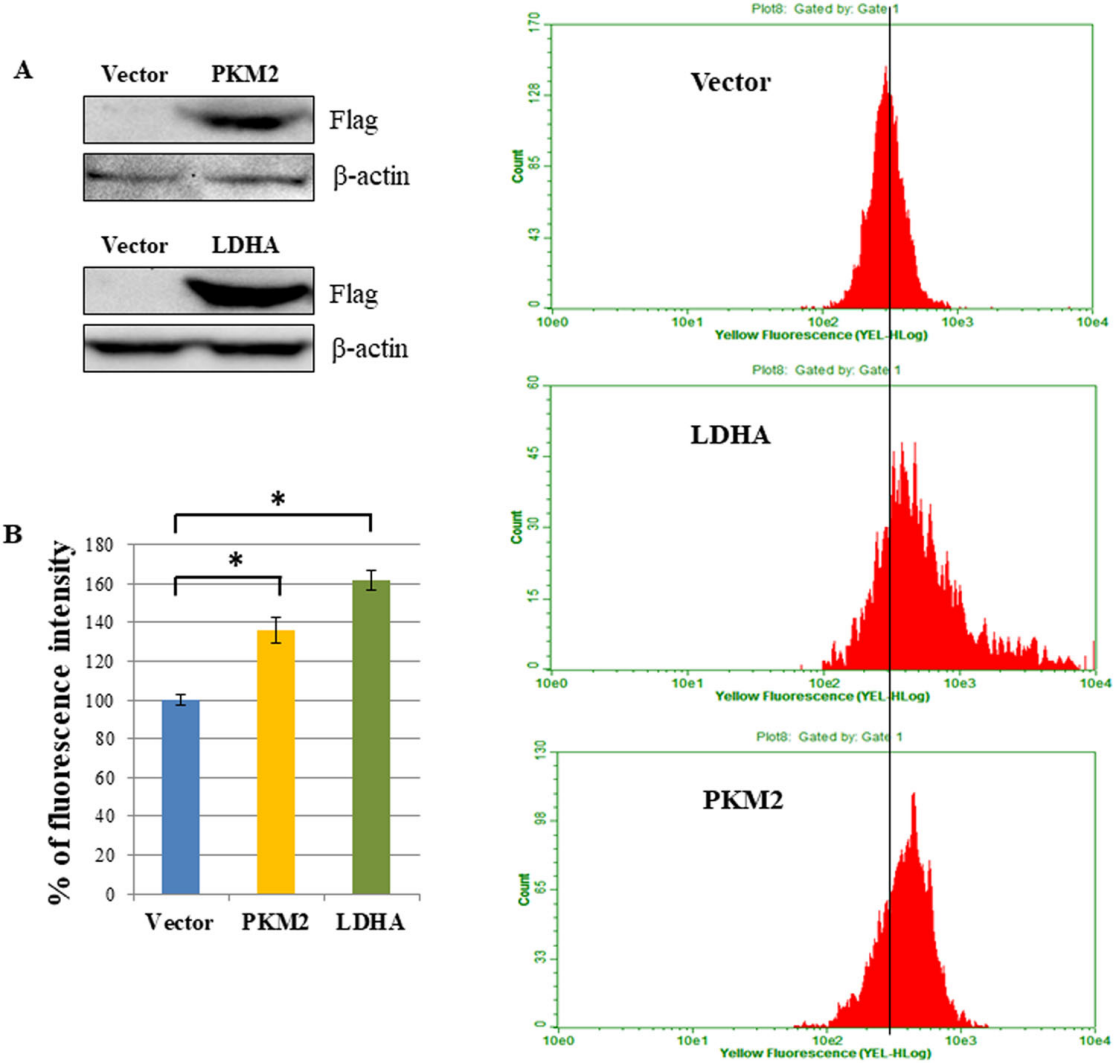
The overexpression of PKM2 and LDHA in H1299 cells increase mitochondrial membrane potential. **A**. Western-blot of H1299 cells transfected by PKM2-encoding and LDHA-encoding vectors. **B**, **C** The flow cytometry of MitoTracker fluorescence of PKM2 and LDHA overexpressing H1299 cells. Three biological replicates were used for all quantifications, data are presented as mean ± S.D., *P < 0.05.

To assess if the elevated levels of PKM2 and LDHA contribute to an increase of the MMP production, we have carried out transient overexpression of Flag-tagged PKM2 and LDHA in H1299 cells (Fig. 8A) followed by staining with MitoTracker and subsequent analysis by flow cytometry.

Figure 8B, C demonstrated that overexpression of either PKM2 or LDHA led to the augmentation of MMP levels up to 138 and 162%, respectively. These data confirm our hypothesis that SEMGs are responsible for the upregulation of energy metabolism, at least in part, through increasing the protein level and activity of PKM2 and LDHA.

## Discussion

Semenogelins 1 and 2 are the two autosomal CTAs, which are most abundant proteins of semen making together up to 40% of all semen proteins. They are synthesized and secreted mainly by the glandular epithelium of the seminal vesicles^22^. According to Lundwall and colleagues, SEMGs mRNA can also be detected in seminal vesicles, seminal ducts, prostate, appendages, trachea, salivary and mammary glands, skin, and macular^8^.

Re-expression of SEMG1 and SEMG2 is frequently observed in prostate cancer^13,23^. Moreover, they were detected in a number of human lung and melanoma cancer cell lines^14^ and patients with NSCLC^24^. Also, SEMGs were found in renal tumors^15^, chronic myelogenous and lymphocytic leukemia and myeloma^12^.

Here, we have shown that SEMGs are frequently expressed at both mRNA and protein levels in different human cell models as well as in clinical samples of lung squamous carcinoma. However, in contrast to the microarray data, SEMG2 was observed at the protein level more often than SEMG1, implying that the latter may be regulated on the post-transcriptional level.

As mentioned earlier, almost all information on the role of SEMGs in reproduction concerns SEMG1, whereas the biological activity of SEMG2 remains largely unknown despite the fact that they share 78% of similarity. Information about the biological activity of SEMGs in cancer is also limited and only describes SEMG1 as a co-activator of androgen receptor in prostate cancer^16^.

In the present study, we sought to identify the PPI of SEMGs by carrying out GST pull-down assay coupled with LC-MS/MS. We have identified 119 proteins, associated with SEMG1 or SEMG2 with only 34 proteins being common binding partners of both SEMG1 and SEMG2. Importantly, 86 interactors were associated specifically with SEMG1 and only four were associated with SEMG2. Thus, both quantitative and functional diversity of interactants indicates a potentially more diverse functional role of SEMG1 in comparison to SEMG2.

Importantly, a large number of SEMG1/2 interactors are represented by RNA processing and splicing factors. One explanation to this is because the RNA binding proteins are very abundant proteins in the cell. Recently, we have shown^25^ that SEMGs displayed either cytoplasmic or nuclear speckle-like localization in different lung adenocarcinoma cell lines. Speckles are small sub-nuclear organelles lacking the outer membrane. They are presumably involved in mediating splicing^26^. Therefore, we concluded that SEMGs may potentially participate in splicing. An additional study of SEMGs subnuclear localization is required.

Among all functional groups of proteins associated with SEMGs, we have focused on two key enzymes of cancer-related metabolism—LDHA and PKM. LDHA greatly contributes to the aerobic glycolysis (Warburg effect)^18,19^, which is associated with aggressive, poor differentiated, metastatic tumors, resistance against chemotherapy, and shortened patients survival^27^. PKM is the critical enzyme for glycolysis^28,29^. PKM2 diverts glucose-derived carbons from catabolic to anabolic (biosynthetic) pathways, which is a hallmark of cancer^30^.

Altered metabolism, including aerobic glycolysis, is a well-known hallmark of malignant cells^31,32,33^. Specific metabolic reprogramming contributes to the adaptation plasticity of cancer cells, which allow them to proliferate, migrate, and combat different stresses^34^. Thus, metabolic differences between tumor and normal cells can be used as a target for novel anticancer strategies^35^.

We found that SEMG1 interacted with both LDHA and PKM2, whereas SEMG1 interacted with PKM2 only. We also showed that the protein level and activity of LDHA and PKM2 were increased in the case of SEMG1 overexpression. In turn, overexpression of SEMG2 elevated the protein level and activity only of PKM2, but not LDHA. These results are in accordance with the LC-MS/MS and co-immunoprecipitation data. A precise molecular mechanism of SEMGs-mediated increase of LDHA and PKM2 activity remains elusive but it possibly includes regulation at the post-translational level.

Further, we have demonstrated that both SEMG1 and SEMG2 upregulate MMP, glycolysis, and respiration. These data are in accordance with SEMGs-mediated increase of the LDHA and PKM2 enzymatic activities because we have also shown that the overexpression of LDHA and PKM2 in our cells led to elevation of MMP. It is important to note that according to the SeaHorse profiling SEMG1 displayed stronger effect on glycolysis and respiration than SEMG2. Possibly, this is due to the SEMG1-mediated augmentation of the LDHA activity in contrast to SEMG2.

We have also demonstrated that both SEMGs increased ROS production up to two times. It contradicts to the previously published data^36^ about ROS scavenging activity of SEMGs in sperm. This discrepancy can be explained at least in part by different backgrounds of cells used in these studies (cancer cells versus sperm, respectively). In our case, SEMGs increased the rate of glycolysis, respiration, and MMP. It is well known that the electron transport chain of mitochondria is the main source of ROS in the cell^37^. In this case, the main production of ROS (superoxide anion) results mainly from two processes: (1) one-electron reduction of oxygen by complex I (NADH/NAD^+^ - reductase); (2) the increase of NADH amount (due to intensification of glycolysis) and a high value of the MMP when the backward electron flow occurs^38,39^. Thus, it can be assumed that the observed SEMGs-mediated increase in the intensities of glycolysis, respiration, and MMP leads to an increase in ROS production.

In line with the previous notion, the tetrameric form of PKM2 was shown to suppress p53 transcriptional activity and apoptosis in the state of high oxidation but enhanced the latter in the low oxidation state. We used MCF-7 cells for pull-down experiments. These cells express wild type p53, which is known to affect many metabolic genes, including LDHA and PKM2^40,41^. To further complicate this situation, it should be noted that p53 is regulated by various post-translational modifications, which in turn, respond to multiple environmental cues^42,43,44^. However, it should be noted that most of our experiments were carried out in cells that either lack p53 (H1299), or bear mutant p53 (MDA-MB-231, Mia-Paca 2). Thus, it is unlikely that the p53 status influences the SEMGs-mediated effects on metabolism.

The biological role of CTAs in both germline tissues and tumors remains poorly understood. Meanwhile, investigation of CTAs biological activity is an important task because germ cells and cells of trophoblast (which normally express CTAs), have much in common with the tumor cells^45,46^. One of the mechanisms of re-expression of CTAs in cancer is linked with specific DNA hypomethylation^47,48^. It is well known that tumor cells, especially low-grade ones, often express a gene pattern similar to embryonic stem cells^46,49^. However, the growing body of evidence suggests that indeed CTAs possess biological properties which favor growth, survival and motility of tumor cells. There are several excellent reviews, for instance^3,45^, which exhaustively describe biological activities of CTAs in neoplastic cells including their effects on proliferation, induction of angiogenesis, genomic instability, tissue invasion and metastasis as well as escape from apoptosis.

Taken together, our data demonstrate that two autosomal CTAs, SEMG1 and SEMG2, are frequently expressed in human malignancies and enhance energy metabolism of cancer cells. To our knowledge, this is the first evidence demonstrating that CTAs can influence on cell metabolism. Further, we demonstrated that the expression of both SEMGs was negatively associated with survival outcomes of patients. Both SEMG1 and SEMG2 were reported to correlate with the survival rate of patients with prostate^13^ and renal^15^ cancers. Taken together, these data indicate towards the predominantly oncogenic features of SEMGs in malignancies.

## Supplementary information

Supplementary Figues Legend

Figure 1 supplement

Figure 2 supplement

Figure 3 supplement

Figure 4 supplement

Figure 5 supplement

Figure 6 supplement

Figure 7 supplement

Supplement_Table 1

Supplement_Table 2

Supplement_Table 3

Supplement_Table 4

Supplement_Table 5

Supplement_Table 6
