# Supplementary material for: SEMG1/2 augment energy metabolism of tumor cells
Source: Cell Death Dis. 2020 Dec 11;11(12):1047. doi: 10.1038/s41419-020-03251-w (PMC7733513; doi:10.1038/s41419-020-03251-w)
Supplement: Supplementary file 1 — Supplementary Figues Legend [file 41419_2020_3251_MOESM1_ESM.docx]

**Supplementary Figures**

***Figure 1S.*** ***The*** **mRNA levels of Semenogelins is higher in tumors than in peritumoral tissues.**

The microarray expression data have been obtained from the open-source database GEO Datasets (GSE31192 – pregnancy-associated breast cancer, GSE116959 - lung adenocarcinoma). Semg1 expression level in breast cancer was significantly higher than in normal tissue (p = 0.015, the Wilcoxon Rank-Sum test).

***Figure 2S.*** ***Semenogelins are not detected in human lung fibroblasts.***

Western blot of two human non-transformed fibroblasts cell lines, DF2 and WI-38, and H520 lung adenocarcinoma cell line assessing the levels of SEMG1 and SEMG2 expression.

**Figure 3S. Proteins associated with both Semenogelins and SEMG1 only.**

Quantitative and functional diversity of proteins associated with both Semenogelins and SEMG1 only.

**Figure 4S. Overexpression of SEMG1 and SEMG2 in MDA-MB-468 cells increases the MMP and ROS production.**

Breast cancer cells MDA-MB-468 were transiently transfected with 3xFlag-tagged SEMG1 and SEMG2, or the corresponding empty vector. Three days after transfection cells were harvested and analyzed for: western blotting for SEMGs’ overexpression (Anti-Flag) (**A**), flow cytometry for MitoTracker staining **(B)**, or DHE (**C-E)** show quantitative results for MitoTracker and DHE fluorescence intensity, respectively.

**Figure 5S. Overexpression of SEMG1 and SEMG2 in MCF7 cells increases the MMP and ROS production.**

Breast cancer cells MCF7 were transiently transfected with 3xFlag-tagged SEMG1 and SEMG2, or the corresponding empty vector. Three days after transfection cells were harvested and analyzed for: western blotting for SEMGs’ overexpression (Anti-Flag) (**A**), flow cytometry for MitoTracker staining **(B)**, or DHE (**C-E)** show quantitative results for MitoTracker and DHE fluorescence intensity, respectively.

**Figure 6S. SEMG1 and SEMG2 up-regulate MMP.**

The confocal microscopy of MitoTracker fluorescence in MDA-MB-231 (**A**) and Mia-Paca 2 (**B**) lines with stable overexpression of SEMG1, SEMG2, or control vector.

**Figure 7S. Knockdowns of SEMG1 and SEMG2 decrease the MMP and ROS production.**

Knockdowns of SEMG1 and SEMG2 by specific shRNAs (Sh_1 and Sh_2) in H520 cells. Western blotting for SEMGs (**A),** flow cytometry of cells stained for MitoTracker (**B),** or DHE (**C)**, (**D)** and (**E)** show quantitative results for MitoTracker and DHE fluorescence intensity, respectively.
