# Supplementary figures and images for: SEMG1/2 augment energy metabolism of tumor cells
Source: Cell Death Dis. 2020 Dec 11;11(12):1047. doi: 10.1038/s41419-020-03251-w (PMC7733513; doi:10.1038/s41419-020-03251-w)

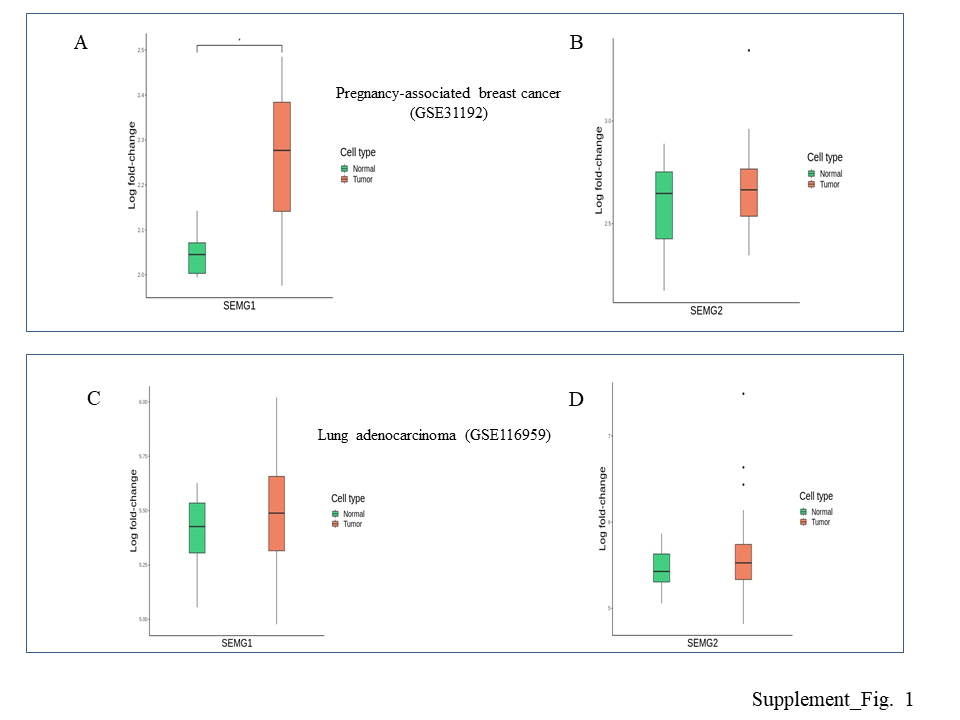

Supplement: Supplementary file 2 — Figure 1 supplement [file 41419_2020_3251_MOESM2_ESM.tif]

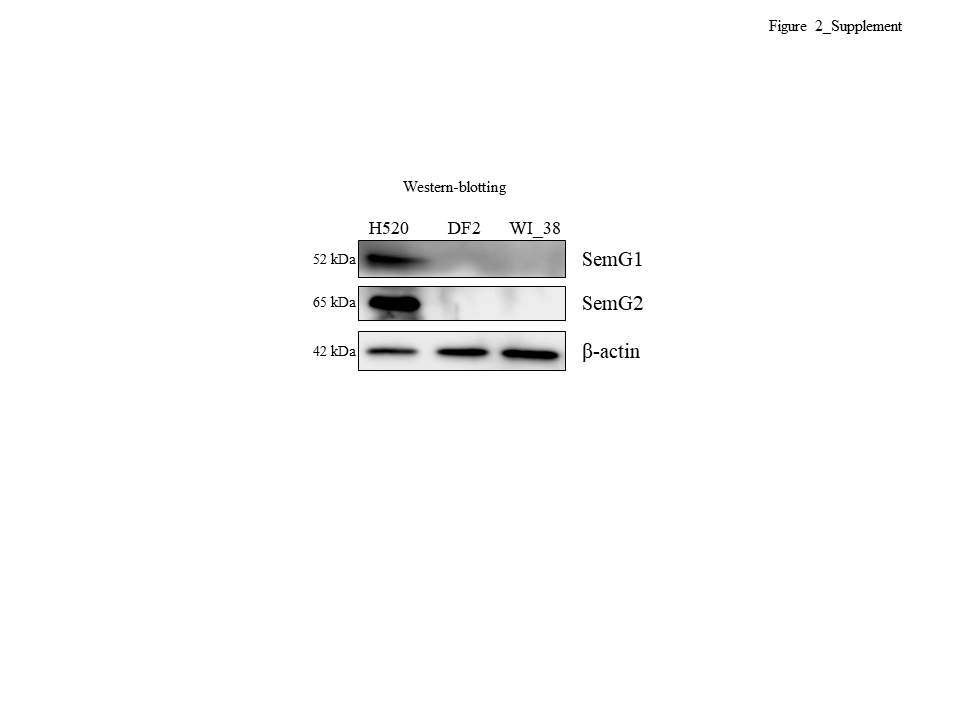

Supplement: Supplementary file 3 — Figure 2 supplement [file 41419_2020_3251_MOESM3_ESM.tif]

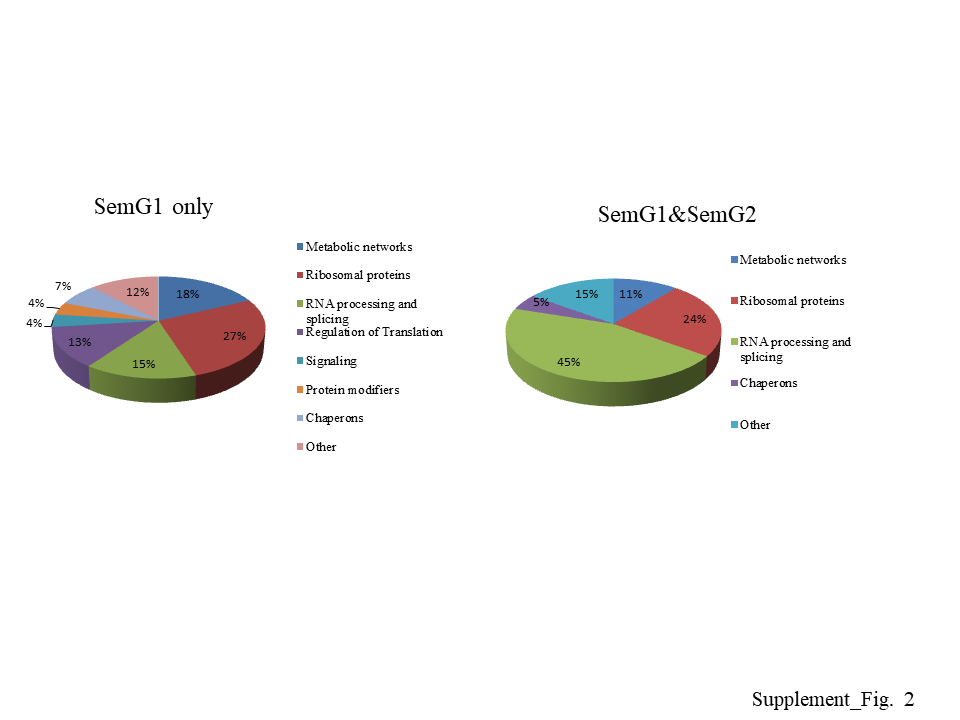

Supplement: Supplementary file 4 — Figure 3 supplement [file 41419_2020_3251_MOESM4_ESM.tif]

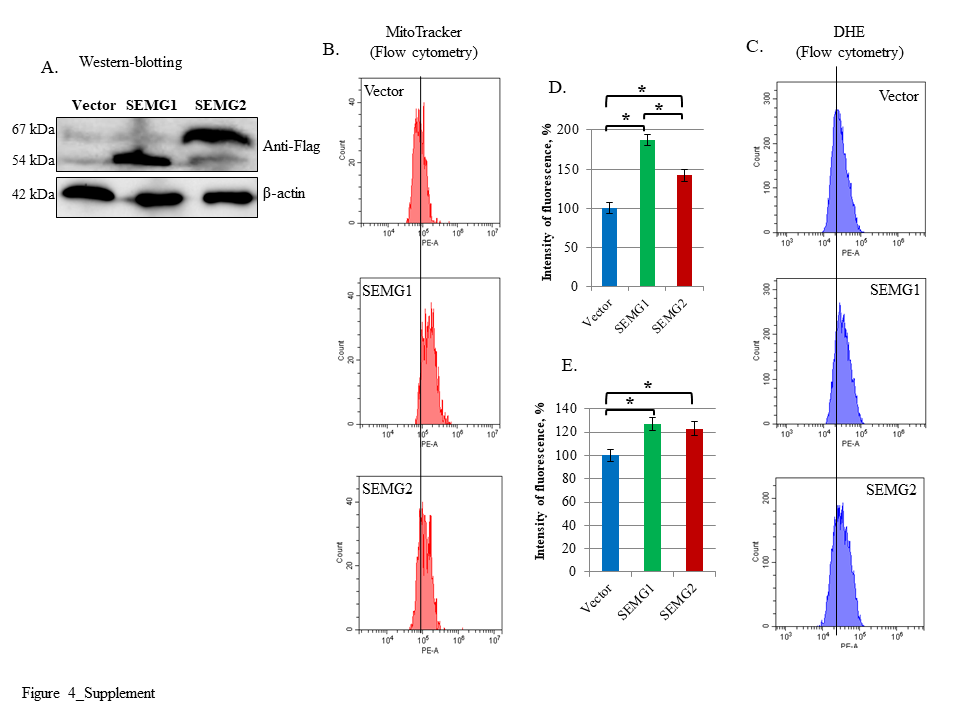

Supplement: Supplementary file 5 — Figure 4 supplement [file 41419_2020_3251_MOESM5_ESM.tif]

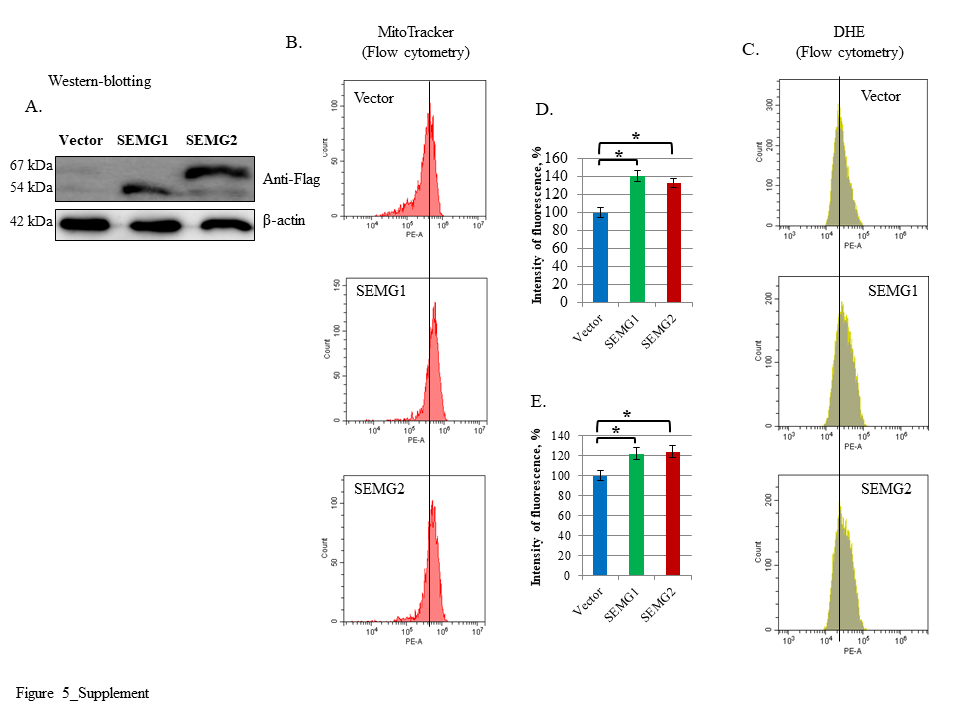

Supplement: Supplementary file 6 — Figure 5 supplement [file 41419_2020_3251_MOESM6_ESM.tif]

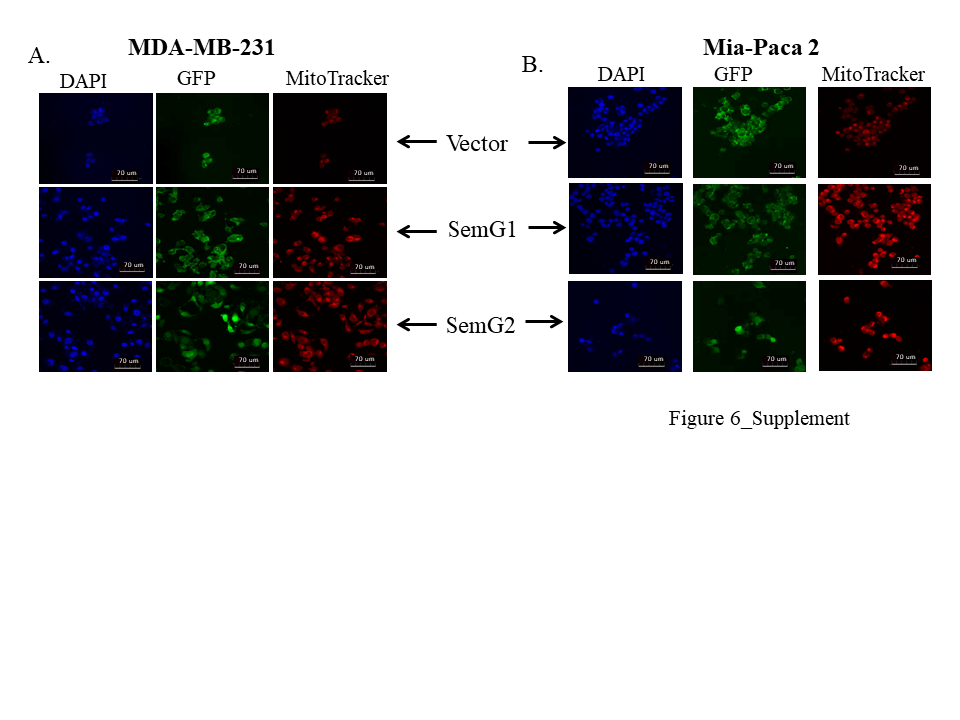

Supplement: Supplementary file 7 — Figure 6 supplement [file 41419_2020_3251_MOESM7_ESM.tif]

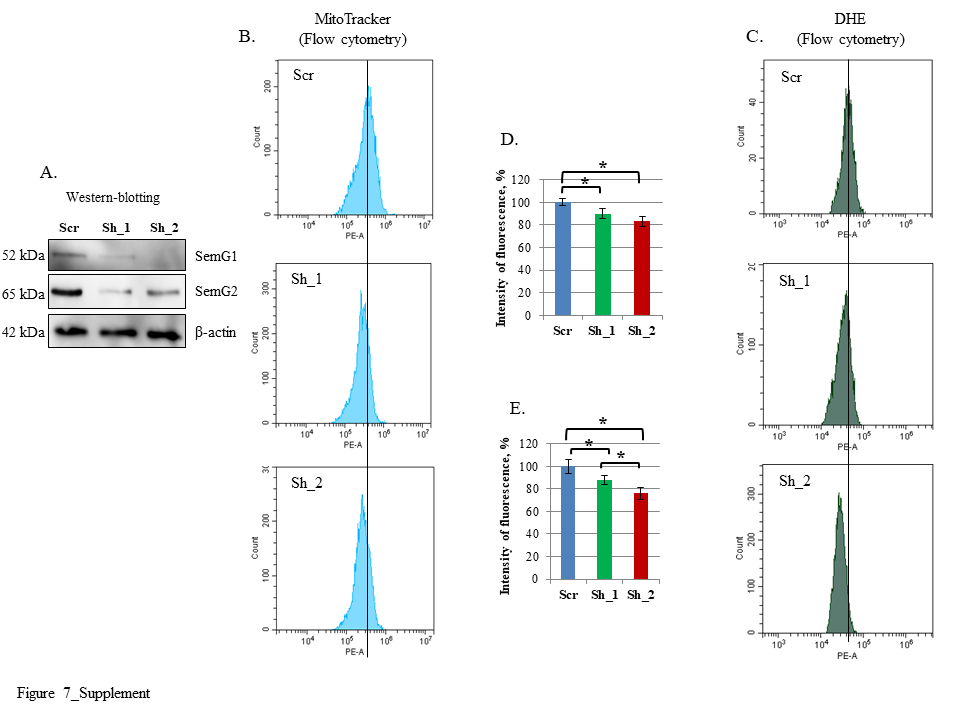

Supplement: Supplementary file 8 — Figure 7 supplement [file 41419_2020_3251_MOESM8_ESM.tif]
