# Supplementary material for: SEMG1/2 augment energy metabolism of tumor cells
Source: Cell Death Dis. 2020 Dec 11;11(12):1047. doi: 10.1038/s41419-020-03251-w (PMC7733513; doi:10.1038/s41419-020-03251-w)
Supplement: Supplementary file 9 — Supplement_Table 1 [file 41419_2020_3251_MOESM9_ESM.pdf]

ALL PROTEINS IDENTIFIED							
				Number of peptides associated with corresponding recombinant protein			
	Identified protein	Accession Number	Molecular weight	GST	GST-SEMG1	GST-SEMG2	
1	PCBP1	Q15365	37 kDa	0	**5**	**4**	Poly(rC)-binding protein 1
2	ALDH18A1	P54886	87 kDa	0	**4**	**0**	Delta-1-pyrroline-5-carboxylate synthase
3	ALYREF	Q86V81	27 kDa	0	**0**	**4**	THO complex subunit 4 OS
4	ATP5A1	P25705	60 kDa	0	**13**	**0**	ATP synthase subunit alpha,
5	ATP5B	H0YH81 (+1)	38 kDa	0	**7**	**0**	ATP synthase subunit beta
6	ATP5O P	P48047	23 kDa	0	**7**	**0**	ATP synthase subunit O, mitochondrial
7	C14orf166	Q9Y224	28 kDa	0	**7**	**0**	UPF0568 protein C14orf166
8	CCT4	P50991	58 kDa	0	**5**	**0**	T-complex protein 1 subunit delta
9	COPA	P53621	138 kDa	0	**6**	**0**	Coatomer subunit alpha
10	CRIP2	H0YFA4 (+1)	21 kDa	0	**3**	**4**	Cysteine-rich protein 2
11	CSDE1	O75534	89 kDa	0	**7**	**3**	Cold shock domain-containing protein E1
12	CYC1	P08574	35 kDa	0	**3**	**0**	Cytochrome c1, heme protein, mitochondrial
13	DDX17	Q92841 (+1)	80 kDa	0	**3**	**2**	Probable ATP-dependent RNA helicase DDX17
14	DDX17	Q92841 [4]	80 kDa	0	**15**	**13**	Probable ATP-dependent RNA helicase DDX17
15	DDX3X	A0A0D9SF53 (+3)	81 kDa	0	**14**	**7**	ATP-dependent RNA helicase DDX3X
16	DDX5	P17844 (+1)	69 kDa	0	**9**	**9**	Probable ATP-dependent RNA helicase DDX5
17	DDX6	P26196	54 kDa	0	**4**	**0**	Probable ATP-dependent RNA helicase DDX6
18	DHX9	Q08211	141 kDa	0	**6**	**0**	ATP-dependent RNA helicase A
19	DRG1	Q9Y295	41 kDa	0	**4**	**0**	Developmentally-regulated GTP-binding protein 1
20	EEF2	P13639	95 kDa	0	**18**	**9**	Elongation factor 2
21	EIF2S1	P05198	36 kDa	0	**4**	**0**	Eukaryotic translation initiation factor 2 subunit 1
22	EIF2S2	P20042	38 kDa	0	**3**	**0**	Eukaryotic translation initiation factor 2 subunit 2
23	EIF2S3	P41091	51 kDa	0	**3**	**0**	Eukaryotic translation initiation factor 2
24	EIF3D	O15371	64 kDa	0	**5**	**0**	Eukaryotic translation initiation factor 3 subunit D
25	EPRS	P07814	171 kDa	0	**6**	**0**	Bifunctional glutamate/proline--tRNA ligase
26	FARSB	Q9NSD9	66 kDa	0	**3**	**0**	Phenylalanine--tRNA ligase beta subunit

27	FASN	A0A0U1RQF0 (+1)	273 kDa	0	**17**	**4**	Fatty acid synthase
28	G3BP1	Q13283	52 kDa	0	**3**	**0**	Ras GTPase-activating protein-binding protein 1
29	hCG_2039566	A0A0U1RR32 (+8)	18 kDa	0	**5**	**0**	Histone H2A
30	HIST1H1T	P22492 [2]	22 kDa	0	**3**	**4**	Cluster of Histone H1t
31	HNRNPA1	F8W6I7 (+1)	33 kDa	0	**8**	**4**	Heterogeneous nuclear ribonucleoprotein A1
32	HNRNPA2B1	P22626	37 kDa	0	**9**	**8**	Heterogeneous nuclear ribonucleoproteins A2/B1
33	HNRNPA3	P51991	40 kDa	0	**4**	**0**	Heterogeneous nuclear ribonucleoprotein A3
34	HNRNPD	H0Y8G5 (+2)	30 kDa	0	**6**	**0**	Heterogeneous nuclear ribonucleoprotein D0
35	HNRNPF	P52597	46 kDa	0	**3**	**0**	Heterogeneous nuclear ribonucleoprotein F
36	HNRNPK	P61978	51 kDa	0	**6**	**0**	Heterogeneous nuclear ribonucleoprotein K
37	HNRNPM	P52272	78 kDa	0	**23**	**15**	Heterogeneous nuclear ribonucleoprotein M
38	HNRNPM	P52272	78 kDa	0	**13**	**5**	Heterogeneous nuclear ribonucleoprotein M
39	HNRNPU	Q00839	91 kDa	0	**20**	**5**	Heterogeneous nuclear ribonucleoprotein U
40	HP1BP3	B0QZK4 (+2)	29 kDa	0	**4**	**0**	Heterochromatin protein 1-binding protein 3
41	HSP90AB1	P08238	83 kDa	0	**11**	**0**	Cluster of Heat shock protein HSP 90-beta
42	HSPA9	P38646	74 kDa	0	**3**	**0**	Stress-70 protein, mitochondrial
43	IDH2	P48735	51 kDa	0	**4**	**0**	Isocitrate dehydrogenase [NADP], mitochondrial
44	KIF5B	P33176	110 kDa	0	**4**	**0**	Kinesin-1 heavy chain
45	LARS	Q9P2J5	134 kDa	0	**3**	**0**	Leucine--tRNA ligase, cytoplasmic
46	LDHA	P00338	37 kDa	0	**5**	**0**	L-lactate dehydrogenase A chain
47	LRRC59	Q96AG4	35 kDa	0	**10**	**5**	Leucine-rich repeat-containing protein 59
48	MARS	P56192	101 kDa	0	**5**	**0**	Methionine--tRNA ligase, cytoplasmic
49	MCM3	J3KQ69 (+1)	92 kDa	0	**3**	**0**	DNA replication licensing factor MCM3
50	MTHFD1	F5H2F4 (+1)	111 kDa	0	**3**	**0**	C-1-tetrahydrofolate synthase, cytoplasmic
51	MYO6	A0A0A0MRM8	145 kDa	0	**5**	**3**	Unconventional myosin-VI
52	NCL	P19338	77 kDa	0	**5**	**4**	Nucleolin
53	NONO	Q15233	54 kDa	0	**3**	**11**	Non-POU domain-containing octamer-binding protein
54	PABPC1	A0A087WTT1 (+2)	59 kDa	0	**4**	**0**	Polyadenylate-binding protein
55	PAICS	E9PBS1 (+1)	46 kDa	0	**3**	**0**	Multifunctional protein ADE2
56	PCBP1	Q15365 [5]	37 kDa	0	**9**	**4**	Cluster of Poly(rC)-binding protein 1
57	PCK2	Q16822	71 kDa	0	**7**	**3**	Phosphoenolpyruvate carboxykinase [GTP], mitochondrial
58	PDIA3	P30101	57 kDa	0	**4**	**8**	Protein disulfide-isomerase A3
59	PDIA6	Q15084	48 kDa	0	**4**	**0**	Protein disulfide-isomerase A6

60	PFKL	P17858	85 kDa	0	**4**	**0**	ATP-dependent 6-phosphofructokinase, liver type
61	PFKM	P08237	85 kDa	0	**3**	**0**	ATP-dependent 6-phosphofructokinase, muscle type
62	PFKP	Q01813	86 kDa	0	**10**	**0**	ATP-dependent 6-phosphofructokinase, platelet type
63	PHB	C9JW96 (+1)	27 kDa	0	**3**	**0**	Prohibitin
64	PKM	P14618	58 kDa	0	**23**	**14**	Pyruvate kinase PKM
65	PPIB	P23284	24 kDa	0	**3**	**0**	Peptidyl-prolyl cis-trans isomerase B
66	PSPC1	Q8WXF1	59 kDa	0	**0**	**5**	Paraspeckle component 1
67	RACK1	P63244	35 kDa	0	**8**	**0**	Receptor of activated protein C kinase 1
68	RARS	P54136	75 kDa	0	**4**	**0**	Arginine--tRNA ligase, cytoplasmic
69	RPL11	P62913	20 kDa	0	**3**	**3**	60S ribosomal protein L11
70	RPL13	P26373	24 kDa	0	**9**	**0**	60S ribosomal protein L13
71	RPL17	A0A087WWH0 (+7	15 kDa	0	**4**	**0**	60S ribosomal protein L17
72	RPL19	J3KTE4 (+2)	23 kDa	0	**4**	**0**	Ribosomal protein L19
73	RPL21	P46778	19 kDa	0	**4**	**0**	60S ribosomal protein L21
74	RPL22	P35268	15 kDa	0	**5**	**0**	60S ribosomal protein L22
75	RPL23	C9JD32 (+2)	10 kDa	0	**5**	**0**	60S ribosomal protein L23
76	RPL23A	P62750	18 kDa	0	**11**	**6**	60S ribosomal protein L23a
77	RPL24	C9JNW5 (+2)	18 kDa	0	**3**	**0**	60S ribosomal protein L24
78	RPL26	P61254 [3]	17 kDa	0	**8**	**6**	Cluster of 60S ribosomal protein L26
79	RPL27A	E9PLL6 (+1)	12 kDa	0	**3**	**0**	60S ribosomal protein L27a
80	RPL29	P47914	18 kDa	0	**0**	**4**	60S ribosomal protein L29
81	RPL31	B7Z4C8 (+4)	15 kDa	0	**5**	**0**	60S ribosomal protein L31
82	RPL35	P42766	15 kDa	0	**5**	**3**	60S ribosomal protein L35
83	RPL4	P36578	48 kDa	0	**4**	**0**	60S ribosomal protein L4
84	RPL9	D6RAN4 (+1)	21 kDa	0	**3**	**0**	60S ribosomal protein L9
85	RPLP0	F8VU65 (+3)	27 kDa	0	**3**	**0**	60S acidic ribosomal protein P0
86	RPS11	P62280	18 kDa	0	**9**	**0**	40S ribosomal protein S11
87	RPS13	P62277	17 kDa	0	**6**	**0**	40S ribosomal protein S13
88	RPS13	P62277	17 kDa	0	**8**	**0**	Cluster of 40S ribosomal protein S13
89	RPS14	P62263	16 kDa	0	**8**	**0**	40S ribosomal protein S14
90	RPS15	K7ELC2 (+2)	18 kDa	0	**4**	**0**	40S ribosomal protein S15
91	RPS17	P08708	16 kDa	0	**6**	**3**	40S ribosomal protein S17
92	RPS18	P62269	18 kDa	0	**11**	**0**	40S ribosomal protein S18

93	RPS2	E9PQD7 (+2)	25 kDa	0	**6**	**0**	40S ribosomal protein S2
94	RPS20	P60866	13 kDa	0	**3**	**0**	40S ribosomal protein S20
95	RPS25	P62851	14 kDa	0	**5**	**0**	40S ribosomal protein S25
96	RPS3	P23396	27 kDa	0	**10**	**0**	40S ribosomal protein S3
97	RPS4X	P62701	30 kDa	0	**9**	**3**	40S ribosomal protein S4, X isoform
98	RPS5	P46782 (+1)	23 kDa	0	**10**	**8**	40S ribosomal protein S5
99	RPS6	P62753	29 kDa	0	**5**	**4**	40S ribosomal protein S6
100	RPS7	P62081	22 kDa	0	**10**	**4**	40S ribosomal protein S7
101	RTCB	Q9Y3I0	55 kDa	0	**7**	**6**	tRNA-splicing ligase RtcB homolog
102	SERBP1	Q8NC51	45 kDa	0	**3**	**0**	Plasminogen activator inhibitor 1 RNA-binding protein
103	SFPQ	P23246	76 kDa	0	**7**	**14**	Splicing factor, proline- and glutamine-rich
104	SHMT2	P34897	56 kDa	0	**8**	**3**	Serine hydroxymethyltransferase, mitochondrial
105	SLC25A5	P05141	33 kDa	0	**5**	**0**	ADP/ATP translocase 2 OS=Homo sapiens
106	SLC3A2	F5GZS6 (+2)	65 kDa	0	**4**	**0**	4F2 cell-surface antigen heavy chain
107	SRSF1	J3KTL2 (+1)	28 kDa	0	**3**	**0**	Serine/arginine-rich-splicing factor 1
108	SRSF2	J3KP15 (+2)	15 kDa	0	**4**	**0**	Serine/arginine-rich-splicing factor 2
109	STRAP	Q9Y3F4	38 kDa	0	**6**	**0**	Serine-threonine kinase receptor-associated protein
110	SYNCRIP	O60506	70 kDa	0	**8**	**2**	Heterogeneous nuclear ribonucleoprotein Q
111	TRIM25	Q14258	71 kDa	0	**3**	**0**	E3 ubiquitin/ISG15 ligase TRIM25
112	TRIM28	Q13263	89 kDa	0	**8**	**0**	Transcription intermediary factor 1-beta
113	TUFM	P49411	50 kDa	0	**11**	**0**	Elongation factor Tu, mitochondrial O
114	UBB	B4DV12 (+16)	17 kDa	0	**3**	**0**	Polyubiquitin-B
115	UFL1	O94874	90 kDa	0	**3**	**0**	E3 UFM1-protein ligase 1
116	USP10	Q14694	87 kDa	0	**3**	**3**	Ubiquitin carboxyl-terminal hydrolase 10
117	XRCC6	B1AHC9 (+1)	64 kDa	0	**4**	**0**	X-ray repair cross-complementing protein 6
118	YBX1	P67809	36 kDa	0	**6**	**0**	Nuclease-sensitive element-binding protein 1
119	ZNF90	A0A087WZ27 (+2)	14 kDa	0	**6**	**0**	Zinc finger protein 90
