# Supplementary material for: SEMG1/2 augment energy metabolism of tumor cells
Source: Cell Death Dis. 2020 Dec 11;11(12):1047. doi: 10.1038/s41419-020-03251-w (PMC7733513; doi:10.1038/s41419-020-03251-w)
Supplement: Supplementary file 10 — Supplement_Table 2 [file 41419_2020_3251_MOESM10_ESM.pdf]

	ALL PROTEINS ASSOCIATED WITH SEMG1								
				Number of peptides associated with corresponding recombinant protein					
	**Identified protein**	**Accession Number**	**Molecular weight**	**GST**	**GST-SEMG1**	**GST-SEMG2**		**Assigned number of protein function**	
1	CYC1	P08574	35 kDa	0	**3**	0		1	Cytochrome c1, heme protein, mitochondrial
2	PAICS	E9PBS1 (+1)	46 kDa	0	**3**	0		1	Multifunctional protein ADE2
3	MTHFD1	F5H2F4 (+1)	111 kDa	0	**3**	0		1	C-1-tetrahydrofolate synthase, cytoplasmic
4	PFKM	P08237	85 kDa	0	**3**	0		1	ATP-dependent 6-phosphofructokinase, muscle type
5	PFKL	P17858	85 kDa	0	**4**	0		1	ATP-dependent 6-phosphofructokinase, liver type
6	SLC3A2	F5GZS6 (+2)	65 kDa	0	**4**	0		1	4F2 cell-surface antigen heavy chain
7	IDH2	P48735	51 kDa	0	**4**	0		1	Isocitrate dehydrogenase [NADP], mitochondrial
8	ALDH18A1	P54886	87 kDa	0	**4**	0		1	Delta-1-pyrroline-5-carboxylate synthase
9	SLC25A5	P05141	33 kDa	0	**5**	0		1	ADP/ATP translocase 2 OS=Homo sapiens
10	LDHA	P00338	37 kDa	0	**5**	0		1	L-lactate dehydrogenase A chain
11	ATP5O P	P48047	23 kDa	0	**7**	0		1	ATP synthase subunit O, mitochondrial
12	ATP5B	H0YH81 (+1)	38 kDa	0	**7**	0		1	ATP synthase subunit beta
13	PCK2	Q16822	71 kDa	0	**7**	3		1	Phosphoenolpyruvate carboxykinase [GTP], mitochondrial
14	SHMT2	P34897	56 kDa	0	**8**	3		1	Serine hydroxymethyltransferase, mitochondrial
15	PFKP	Q01813	86 kDa	0	**10**	0		1	ATP-dependent 6-phosphofructokinase, platelet type
16	ATP5A1	P25705	60 kDa	0	**13**	0		1	ATP synthase subunit alpha,
17	FASN	A0A0U1RQF0 (+1)	273 kDa	0	**17**	4		1	Fatty acid synthase
18	PKM	P14618	58 kDa	0	**23**	14		1	Pyruvate kinase PKM
19	RPL27A	E9PLL6 (+1)	12 kDa	0	**3**	0		2	60S ribosomal protein L27a
20	RPS20	P60866	13 kDa	0	**3**	0		2	40S ribosomal protein S20
21	RPL24	C9JNW5 (+2)	18 kDa	0	**3**	0		2	60S ribosomal protein L24
22	RPL9	D6RAN4 (+1)	21 kDa	0	**3**	0		2	60S ribosomal protein L9

23	RPLP0	F8VU65 (+3)	27 kDa	0	**3**	0		2	60S acidic ribosomal protein P0
24	RPL11	P62913	20 kDa	0	**3**	3		2	60S ribosomal protein L11
25	RPL21	P46778	19 kDa	0	**4**	0		2	60S ribosomal protein L21
26	RPL17	A0A087WWH0 (+7)	15 kDa	0	**4**	0		2	60S ribosomal protein L17
27	RPL19	J3KTE4 (+2)	23 kDa	0	**4**	0		2	Ribosomal protein L19
28	RPS15	K7ELC2 (+2)	18 kDa	0	**4**	0		2	40S ribosomal protein S15
29	RPL4	P36578	48 kDa	0	**4**	0		2	60S ribosomal protein L4
30	RPS25	P62851	14 kDa	0	**5**	0		2	40S ribosomal protein S25
31	RPL22	P35268	15 kDa	0	**5**	0		2	60S ribosomal protein L22
32	RPL31	B7Z4C8 (+4)	15 kDa	0	**5**	0		2	60S ribosomal protein L31
33	RPL23	C9JD32 (+2)	10 kDa	0	**5**	0		2	60S ribosomal protein L23
34	RPL35	P42766	15 kDa	0	**5**	3		2	60S ribosomal protein L35
35	RPS6	P62753	29 kDa	0	**5**	4		2	40S ribosomal protein S6
36	RPS13	P62277	17 kDa	0	**6**	0		2	40S ribosomal protein S13
37	RPS2	E9PQD7 (+2)	25 kDa	0	**6**	0		2	40S ribosomal protein S2
38	RPS17	P08708	16 kDa	0	**6**	3		2	40S ribosomal protein S17
39	RPS13	P62277	17 kDa	0	**8**	0		2	Cluster of 40S ribosomal protein S13
40	RPS14	P62263	16 kDa	0	**8**	0		2	40S ribosomal protein S14
41	RPL26	P61254 [3]	17 kDa	0	**8**	6		2	Cluster of 60S ribosomal protein L26
42	RPL13	P26373	24 kDa	0	**9**	0		2	60S ribosomal protein L13
43	RPS11	P62280	18 kDa	0	**9**	0		2	40S ribosomal protein S11
44	RPS4X	P62701	30 kDa	0	**9**	3		2	40S ribosomal protein S4, X isoform
45	RPS3	P23396	27 kDa	0	**10**	0		2	40S ribosomal protein S3
46	RPS7	P62081	22 kDa	0	**10**	4		2	40S ribosomal protein S7
47	RPS5	P46782 (+1)	23 kDa	0	**10**	8		2	40S ribosomal protein S5
48	RPS18	P62269	18 kDa	0	**11**	0		2	40S ribosomal protein S18
49	RPL23A	P62750	18 kDa	0	**11**	6		2	60S ribosomal protein L23a
50	SERBP1	Q8NC51	45 kDa	0	**3**	0		3	Plasminogen activator inhibitor 1 RNA-binding protein
51	HNRNPF	P52597	46 kDa	0	**3**	0		3	Heterogeneous nuclear ribonucleoprotein F
52	SRSF1	J3KTL2 (+1)	28 kDa	0	**3**	0		3	Serine/arginine-rich-splicing factor 1
53	DDX17	Q92841 (+1)	80 kDa	0	**3**	2		3	Probable ATP-dependent RNA helicase DDX17
54	NONO	Q15233	54 kDa	0	**3**	11		3	Non-POU domain-containing octamer-binding protein
55	HP1BP3	B0QZK4 (+2)	29 kDa	0	**4**	0		3	Heterochromatin protein 1-binding protein 3
56	HNRNPA3	P51991	40 kDa	0	**4**	0		3	Heterogeneous nuclear ribonucleoprotein A3
57	PABPC1	A0A087WTT1 (+2)	59 kDa	0	**4**	0		3	Polyadenylate-binding protein
58	SRSF2	J3KP15 (+2)	15 kDa	0	**4**	0		3	Serine/arginine-rich-splicing factor 2

59	DDX6	P26196	54 kDa	0	**4**	0		3	Probable ATP-dependent RNA helicase DDX6
60	PCBP1	Q15365	37 kDa	0	**5**	4		3	Poly(rC)-binding protein 1
61	YBX1	P67809	36 kDa	0	**6**	0		3	Nuclease-sensitive element-binding protein 1
62	HNRNPK	P61978	51 kDa	0	**6**	0		3	Heterogeneous nuclear ribonucleoprotein K
63	HNRNPD	H0Y8G5 (+2)	30 kDa	0	**6**	0		3	Heterogeneous nuclear ribonucleoprotein D0
64	DHX9	Q08211	141 kDa	0	**6**	0		3	ATP-dependent RNA helicase A
65	RTCB	Q9Y3I0	55 kDa	0	**7**	6		3	tRNA-splicing ligase RtcB homolog
66	SFPQ	P23246	76 kDa	0	**7**	14		3	Splicing factor, proline- and glutamine-rich
67	SYNCRIP	O60506	70 kDa	0	**8**	2		3	Heterogeneous nuclear ribonucleoprotein Q
68	HNRNPA1	F8W6I7 (+1)	33 kDa	0	**8**	4		3	Heterogeneous nuclear ribonucleoprotein A1
69	PCBP1	Q15365 [5]	37 kDa	0	**9**	4		3	Cluster of Poly(rC)-binding protein 1
70	HNRNPA2B1	P22626	37 kDa	0	**9**	8		3	Heterogeneous nuclear ribonucleoproteins A2/B1
71	DDX5	P17844 (+1)	69 kDa	0	**9**	9		3	Probable ATP-dependent RNA helicase DDX5
72	DDX1	Q92499	82 kDa	0	**11**	3		3	ATP-dependent RNA helicase DDX1
73	HNRNPM	P52272	78 kDa	0	**13**	5		3	Heterogeneous nuclear ribonucleoprotein M
74	DDX3X	A0A0D9SF53 (+3)	81 kDa	0	**14**	7		3	ATP-dependent RNA helicase DDX3X
75	DDX17	Q92841 [4]	80 kDa	0	**15**	13		3	Probable ATP-dependent RNA helicase DDX17
76	HNRNPU	Q00839	91 kDa	0	**20**	5		3	Heterogeneous nuclear ribonucleoprotein U
77	HNRNPM	P52272	78 kDa	0	**23**	15		3	Heterogeneous nuclear ribonucleoprotein M
78	HSPA9	P38646	74 kDa	0	**3**	0		4	Stress-70 protein, mitochondrial
79	PPIB	P23284	24 kDa	0	**3**	0		4	Peptidyl-prolyl cis-trans isomerase B
80	PDIA6	Q15084	48 kDa	0	**4**	0		4	Protein disulfide-isomerase A6
81	PDIA3	P30101	57 kDa	0	**4**	8		4	Protein disulfide-isomerase A3
82	CCT4	P50991	58 kDa	0	**5**	0		4	T-complex protein 1 subunit delta
83	CSDE1	O75534	89 kDa	0	**7**	3		4	Cold shock domain-containing protein E1
84	HSP90AB1	P08238	83 kDa	0	**11**	0		4	Cluster of Heat shock protein HSP 90-beta

85	G3BP1	Q13283	52 kDa	0	**3**	0		6	Ras GTPase-activating protein-binding protein 1
86	STRAP	Q9Y3F4	38 kDa	0	**6**	0		6	Serine-threonine kinase receptor-associated protein
87	RACK1	P63244	35 kDa	0	**8**	0		6	Receptor of activated protein C kinase 1
88	UBB	B4DV12 (+16)	17 kDa	0	**3**	0		11	Polyubiquitin-B
89	PHB	C9JW96 (+1)	27 kDa	0	**3**	0		6	Prohibitin
90	MCM3	J3KQ69 (+1)	92 kDa	0	**3**	0		6	DNA replication licensing factor MCM3
91	HIST1H1T	P22492 [2]	22 kDa	0	**3**	4		6	Cluster of Histone H1t
92	CRIP2	H0YFA4 (+1)	21 kDa	0	**3**	4		6	Cysteine-rich protein 2
93	XRCC6	B1AHC9 (+1)	64 kDa	0	**4**	0		6	X-ray repair cross-complementing protein 6
94	DRG1	Q9Y295	41 kDa	0	**4**	0		6	Developmentally-regulated GTP-binding protein 1
95	hCG_2039566	A0A0U1RR32 (+8)	18 kDa	0	**5**	0		6	Histone H2A
96	ZNF90	A0A087WZ27 (+2)	14 kDa	0	**6**	0		6	Zinc finger protein 90
97	C14orf166	Q9Y224	28 kDa	0	**7**	0		6	UPF0568 protein C14orf166
98	TRIM28	Q13263	89 kDa	0	**8**	0		6	Transcription intermediary factor 1-beta
99	LRRC59	Q96AG4	35 kDa	0	**10**	5		6	Leucine-rich repeat-containing protein 59
100	EIF2S3	P41091	51 kDa	0	**3**	0		8	Eukaryotic translation initiation factor 2
101	LARS	Q9P2J5	134 kDa	0	**3**	0		8	Leucine--tRNA ligase, cytoplasmic
102	EIF2S2	P20042	38 kDa	0	**3**	0		8	Eukaryotic translation initiation factor 2 subunit 2
103	FARSB	Q9NSD9	66 kDa	0	**3**	0		8	Phenylalanine--tRNA ligase beta subunit
104	EIF2S1	P05198	36 kDa	0	**4**	0		8	Eukaryotic translation initiation factor 2 subunit 1
105	RARS	P54136	75 kDa	0	**4**	0		8	Arginine--tRNA ligase, cytoplasmic
106	EIF3D	O15371	64 kDa	0	**5**	0		8	Eukaryotic translation initiation factor 3 subunit D
107	MARS	P56192	101 kDa	0	**5**	0		8	Methionine--tRNA ligase, cytoplasmic
108	NCL	P19338	77 kDa	0	**5**	4		8	Nucleolin
109	EPRS	P07814	171 kDa	0	**6**	0		8	Bifunctional glutamate/proline--tRNA ligase
110	TUFM	P49411	50 kDa	0	**11**	0		8	Elongation factor Tu, mitochondrial O
111	EEF2	P13639	95 kDa	0	**18**	9		8	Elongation factor 2
112	KIF5B	P33176	110 kDa	0	**4**	0		9	Kinesin-1 heavy chain
113	MYO6	A0A0A0MRM8	145 kDa	0	**5**	3		9	Unconventional myosin-VI
114	COPA	P53621	138 kDa	0	**6**	0		9	Coatomer subunit alpha
115	TRIM25	Q14258	71 kDa	0	**3**	0		11	E3 ubiquitin/ISG15 ligase TRIM25

116	UFL1	O94874	90 kDa	0	**3**	0		11	E3 UFM1-protein ligase 1
117	USP10	Q14694	87 kDa	0	**3**	3		11	Ubiquitin carboxyl-terminal hydrolase 10

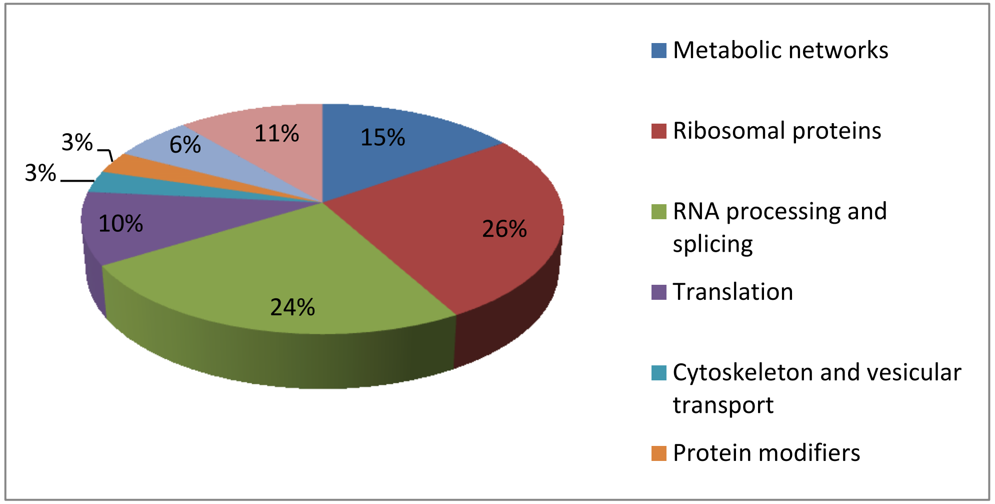

Assigned number of protein function	Protein function	Number of proteins identified	Percentage
1	***Metabolic networks***	18	**15**
2	***Ribosomal proteins***	31	**26**
3	***RNA processing and splicing***	28	**24**
8	***Translation***	12	**10**
9	***Cytoskeleton and vesicular transport***	3	**3**
11	***Protein modifiers***	4	**3**
4	***Chaperons***	7	**6**
6	***Other***	14	**11**
