# Supplementary material for: SEMG1/2 augment energy metabolism of tumor cells
Source: Cell Death Dis. 2020 Dec 11;11(12):1047. doi: 10.1038/s41419-020-03251-w (PMC7733513; doi:10.1038/s41419-020-03251-w)
Supplement: Supplementary file 11 — Supplement_Table 3 [file 41419_2020_3251_MOESM11_ESM.pdf]

ALL PROTEINS ASSOCIATED WITH SEMG2

				Number of peptides associated with corresponding recombinant protein			
				GST	GST-SEMG1	GST-SEMG2	Assigned number of protein function
Fatty acid synthase	FASN	A0A0U1RQF0 (+1)	273 kDa	0	17	4	1
Phosphoenolpyruvate carboxykinase [GTP], mitochondrial	PCK2	Q16822	71 kDa	0	7	**3**	1
Pyruvate kinase PKM	PKM	P14618	58 kDa	0	23	**14**	1
Serine hydroxymethyltransferase, mitochondrial	SHMT2	P34897	56 kDa	0	8	**3**	1
40S ribosomal protein S17	RPS17	P08708	16 kDa	0	6	**3**	2
40S ribosomal protein S4, X isoform	RPS4X	P62701	30 kDa	0	9	**3**	2
40S ribosomal protein S5	RPS5	P46782 (+1)	23 kDa	0	10	**8**	2
40S ribosomal protein S6	RPS6	P62753	29 kDa	0	5	**4**	2
40S ribosomal protein S7	RPS7	P62081	22 kDa	0	10	**4**	2
60S ribosomal protein L11	RPL11	P62913	20 kDa	0	3	**3**	2
60S ribosomal protein L23a	RPL23A	P62750	18 kDa	0	11	**6**	2
60S ribosomal protein L29	RPL29	P47914	18 kDa	0	0	**4**	2
60S ribosomal protein L35	RPL35	P42766	15 kDa	0	5	**3**	2
Cluster of 60S ribosomal protein L26	RPL26	P61254 [3]	17 kDa	0	8	**6**	2
Heterogeneous nuclear ribonucleoprotein A1	HNRNPA1	F8W6I7 (+1)	33 kDa	0	8	**4**	3
Heterogeneous nuclear ribonucleoprotein M	HNRNPM	P52272	78 kDa	0	13	**5**	3
Poly(rC)-binding protein 1	PCBP1	Q15365	37 kDa	0	5	**4**	3
Probable ATP-dependent RNA helicase DDX17	DDX17	Q92841 (+1)	80 kDa	0	3	**2**	3
Probable ATP-dependent RNA helicase DDX5	DDX5	P17844 (+1)	69 kDa	0	9	**9**	3
Heterogeneous nuclear ribonucleoprotein M	HNRNPM	P52272	78 kDa	0	23	**15**	3
Probable ATP-dependent RNA helicase DDX17	DDX17	Q92841 [4]	80 kDa	0	15	**13**	3
ATP-dependent RNA helicase DDX1	DDX1	Q92499	82 kDa	0	11	**3**	3
ATP-dependent RNA helicase DDX3X	DDX3X	A0A0D9SF53 (+3)	81 kDa	0	14	**7**	3
Cluster of Poly(rC)-binding protein 1	PCBP1	Q15365 [5]	37 kDa	0	9	**4**	3

Heterogeneous nuclear ribonucleoprotein Q	SYNCRIP	O60506	70 kDa	0	8	**2**	3
Heterogeneous nuclear ribonucleoprotein U	HNRNPU	Q00839	91 kDa	0	20	**5**	3
Heterogeneous nuclear ribonucleoproteins A2/B1	HNRNPA2B1	P22626	37 kDa	0	9	**8**	3
Non-POU domain-containing octamer-binding protein	NONO	Q15233	54 kDa	0	3	**11**	3
Paraspeckle component 1	PSPC1	Q8WXF1	59 kDa	0	0	**5**	3
Splicing factor, proline- and glutamine-rich	SFPQ	P23246	76 kDa	0	7	**14**	3
tRNA-splicing ligase RtcB homolog	RTCB	Q9Y3I0	55 kDa	0	7	**6**	3
Cold shock domain-containing protein E1	CSDE1	O75534	89 kDa	0	7	**3**	4
Protein disulfide-isomerase A3	PDIA3	P30101	57 kDa	0	4	**8**	4
THO complex subunit 4 OS	ALYREF	Q86V81	27 kDa	0	0	**4**	4
Cluster of Histone H1t	HIST1H1T	P22492 [2]	22 kDa	0	3	**4**	6
Cysteine-rich protein 2	CRIP2	H0YFA4 (+1)	21 kDa	0	3	**4**	6
Leucine-rich repeat-containing protein 59	LRRC59	Q96AG4	35 kDa	0	10	**5**	6
Elongation factor 2	EEF2	P13639	95 kDa	0	18	**9**	6
Nucleolin	NCL	P19338	77 kDa	0	5	**4**	6
Unconventional myosin-VI	MYO6	A0A0A0MR M8	145 kDa	0	5	**3**	6
Ubiquitin carboxyl-terminal hydrolase 10	USP10	Q14694	87 kDa	0	3	**3**	6

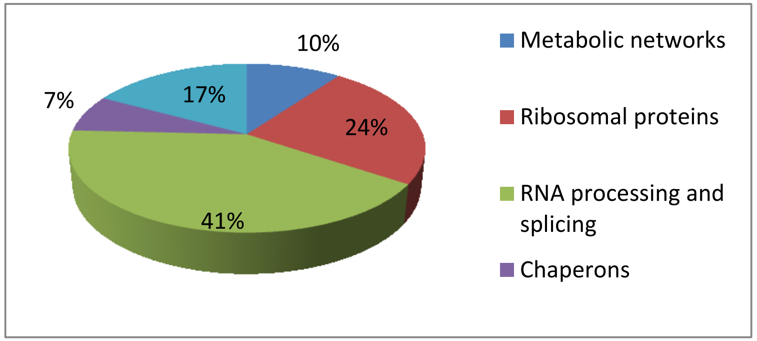

Assigned number of protein function	Protein function	Number of proteins identified	Percentage
1	Metabolic networks	4	**10**
2	Ribosomal proteins	10	**24**
3	RNA processing and splicing	17	**41**
4	Chaperons	3	**7**
6	Other	7	**17**
