# Supplementary material for: SEMG1/2 augment energy metabolism of tumor cells
Source: Cell Death Dis. 2020 Dec 11;11(12):1047. doi: 10.1038/s41419-020-03251-w (PMC7733513; doi:10.1038/s41419-020-03251-w)

PROTEINS ASSOCIATED ONLY WITH SEMG1							
			Number of peptides associated with corresponding recombinant protein				
Identified protein	Accession Number	Molecular weight	GST	GST-SEMG1	GST-SEMG2	Assigned number of protein function	
CYC1	P08574	35 kDa	0	**3**	0	1	Cytochrome c1, heme protein, mitochondrial
PAICS	E9PBS1 (+1)	46 kDa	0	**3**	0	1	Multifunctional protein ADE2
MTHFD1	F5H2F4 (+1)	111 kDa	0	**3**	0	1	C-1-tetrahydrofolate synthase, cytoplasmic
PFKM	P08237	85 kDa	0	**3**	0	1	ATP-dependent 6-phosphofructokinase, muscle type
PFKL	P17858	85 kDa	0	**4**	0	1	ATP-dependent 6-phosphofructokinase, liver type
SLC3A2	F5GZS6 (+2)	65 kDa	0	**4**	0	1	4F2 cell-surface antigen heavy chain
IDH2	P48735	51 kDa	0	**4**	0	1	Isocitrate dehydrogenase [NADP], mitochondrial
ALDH18A1	P54886	87 kDa	0	**4**	0	1	Delta-1-pyrroline-5-carboxylate synthase
SLC25A5	P05141	33 kDa	0	**5**	0	1	ADP/ATP translocase 2 OS=Homo sapiens
LDHA	P00338	37 kDa	0	**5**	0	1	L-lactate dehydrogenase A chain
ATP5O P	P48047	23 kDa	0	**7**	0	1	ATP synthase subunit O, mitochondrial
ATP5B	H0YH81 (+1)	38 kDa	0	**7**	0	1	ATP synthase subunit beta
PFKP	Q01813	86 kDa	0	**10**	0	1	ATP-dependent 6-phosphofructokinase, platelet type

ATP5A1	P25705	60 kDa	0	**13**	0	1	ATP synthase subunit alpha,
RPL27A	E9PLL6 (+1)	12 kDa	0	**3**	0	2	60S ribosomal protein L27a
RPS20	P60866	13 kDa	0	**3**	0	2	40S ribosomal protein S20
RPL24	C9JNW5 (+2)	18 kDa	0	**3**	0	2	60S ribosomal protein L24
RPL9	D6RAN4 (+1)	21 kDa	0	**3**	0	2	60S ribosomal protein L9
RPLP0	F8VU65 (+3)	27 kDa	0	**3**	0	2	60S acidic ribosomal protein P0
RPL21	P46778	19 kDa	0	**4**	0	2	60S ribosomal protein L21
RPL17	A0A087WWH0 (+7)	15 kDa	0	**4**	0	2	60S ribosomal protein L17
RPL19	J3KTE4 (+2)	23 kDa	0	**4**	0	2	Ribosomal protein L19
RPS15	K7ELC2 (+2)	18 kDa	0	**4**	0	2	40S ribosomal protein S15
RPL4	P36578	48 kDa	0	**4**	0	2	60S ribosomal protein L4
RPS25	P62851	14 kDa	0	**5**	0	2	40S ribosomal protein S25
RPL22	P35268	15 kDa	0	**5**	0	2	60S ribosomal protein L22
RPL31	B7Z4C8 (+4)	15 kDa	0	**5**	0	2	60S ribosomal protein L31
RPL23	C9JD32 (+2)	10 kDa	0	**5**	0	2	60S ribosomal protein L23
RPS13	P62277	17 kDa	0	**6**	0	2	40S ribosomal protein S13
RPS2	E9PQD7 (+2)	25 kDa	0	**6**	0	2	40S ribosomal protein S2
RPS13	P62277	17 kDa	0	**8**	0	2	Cluster of 40S ribosomal protein S13

RPS14	P62263	16 kDa	0	**8**	0	2	40S ribosomal protein S14
RPL13	P26373	24 kDa	0	**9**	0	2	60S ribosomal protein L13
RPS11	P62280	18 kDa	0	**9**	0	2	40S ribosomal protein S11
RPS3	P23396	27 kDa	0	**10**	0	2	40S ribosomal protein S3
RPS18	P62269	18 kDa	0	**11**	0	2	40S ribosomal protein S18
SERBP1	Q8NC51	45 kDa	0	**3**	0	3	Plasminogen activator inhibitor 1 RNA-binding protein
HNRNPF	P52597	46 kDa	0	**3**	0	3	Heterogeneous nuclear ribonucleoprotein F
SRSF1	J3KTL2 (+1)	28 kDa	0	**3**	0	3	Serine/arginine-rich-splicing factor 1
HP1BP3	B0QZK4 (+2)	29 kDa	0	**4**	0	3	Heterochromatin protein 1-binding protein 3
HNRNPA3	P51991	40 kDa	0	**4**	0	3	Heterogeneous nuclear ribonucleoprotein A3
PABPC1	A0A087WTT1 (+2)	59 kDa	0	**4**	0	3	Polyadenylate-binding protein
SRSF2	J3KP15 (+2)	15 kDa	0	**4**	0	3	Serine/arginine-rich-splicing factor 2
DDX6	P26196	54 kDa	0	**4**	0	3	Probable ATP-dependent RNA helicase DDX6
YBX1	P67809	36 kDa	0	**6**	0	3	Nuclease-sensitive element-binding protein 1
HNRNPK	P61978	51 kDa	0	**6**	0	3	Heterogeneous nuclear ribonucleoprotein K
HNRNPD	H0Y8G5 (+2)	30 kDa	0	**6**	0	3	Heterogeneous nuclear ribonucleoprotein D0
DHX9	Q08211	141 kDa	0	**6**	0	3	ATP-dependent RNA helicase A
HSPA9	P38646	74 kDa	0	**3**	0	4	Stress-70 protein, mitochondrial

PPIB	P23284	24 kDa	0	**3**	0	4	Peptidyl-prolyl cis-trans isomerase B
PDIA6	Q15084	48 kDa	0	**4**	0	4	Protein disulfide-isomerase A6
CCT4	P50991	58 kDa	0	**5**	0	4	T-complex protein 1 subunit delta
HSP90AB1	P08238	83 kDa	0	**10**	0	4	Heat shock protein HSP 90-beta
HSP90AB1	P08238	83 kDa	0	**11**	0	4	Cluster of Heat shock protein HSP 90-beta
G3BP1	Q13283	52 kDa	0	**3**	0	5	Ras GTPase-activating protein-binding protein 1
STRAP	Q9Y3F4	38 kDa	0	**6**	0	5	Serine-threonine kinase receptor-associated protein
RACK1	P63244	35 kDa	0	**8**	0	5	Receptor of activated protein C kinase 1
UBB	B4DV12 (+16)	17 kDa	0	**3**	0	7	Polyubiquitin-B
PHB	C9JW96 (+1)	27 kDa	0	**3**	0	6	Prohibitin
MCM3	J3KQ69 (+1)	92 kDa	0	**3**	0	6	DNA replication licensing factor MCM3
XRCC6	B1AHC9 (+1)	64 kDa	0	**4**	0	6	X-ray repair cross-complementing protein 6
DRG1	Q9Y295	41 kDa	0	**4**	0	6	Developmentally-regulated GTP-binding protein 1
hCG_2039566	A0A0U1RR32 (+8)	18 kDa	0	**5**	0	6	Histone H2A
ZNF90	A0A087WZ27 (+2)	14 kDa	0	**6**	0	6	Zinc finger protein 90
C14orf166	Q9Y224	28 kDa	0	**7**	0	6	UPF0568 protein C14orf166
TRIM28	Q13263	89 kDa	0	**8**	0	6	Transcription intermediary factor 1-beta
EIF2S3	P41091	51 kDa	0	**3**	0	8	Eukaryotic translation initiation factor 2

LARS	Q9P2J5	134 kDa	0	**3**	0	8	Leucine--tRNA ligase, cytoplasmic
EIF2S2	P20042	38 kDa	0	**3**	0	8	Eukaryotic translation initiation factor 2 subunit 2
FARSB	Q9NSD9	66 kDa	0	**3**	0	8	Phenylalanine--tRNA ligase beta subunit
EIF2S1	P05198	36 kDa	0	**4**	0	8	Eukaryotic translation initiation factor 2 subunit 1
RARS	P54136	75 kDa	0	**4**	0	8	Arginine--tRNA ligase, cytoplasmic
EIF3D	O15371	64 kDa	0	**5**	0	8	Eukaryotic translation initiation factor 3 subunit D
MARS	P56192	101 kDa	0	**5**	0	8	Methionine--tRNA ligase, cytoplasmic
EPRS	P07814	171 kDa	0	**6**	0	8	Bifunctional glutamate/proline--tRNA ligase
TUFM	P49411	50 kDa	0	**11**	0	8	Elongation factor Tu, mitochondrial O
KIF5B	P33176	110 kDa	0	**4**	0	6	Kinesin-1 heavy chain
COPA	P53621	138 kDa	0	**6**	0	6	Coatomer subunit alpha
TRIM25	Q14258	71 kDa	0	**3**	0	7	E3 ubiquitin/ISG15 ligase TRIM25
UFL1	O94874	90 kDa	0	**3**	0	7	E3 UFM1-protein ligase 1

Assigned number of protein function	Protein function	Number of proteins identified	Percentage
1	Metabolic networks	14	**18**
2	Ribosomal proteins	22	**27**
3	RNA processing and splicing	12	**15**
8	Regulation of Translation	10	**13**
5	Signaling	3	**4**
7	Protein modifiers	3	**4**
4	Chaperons	6	**7**
6	Other	10	**12**

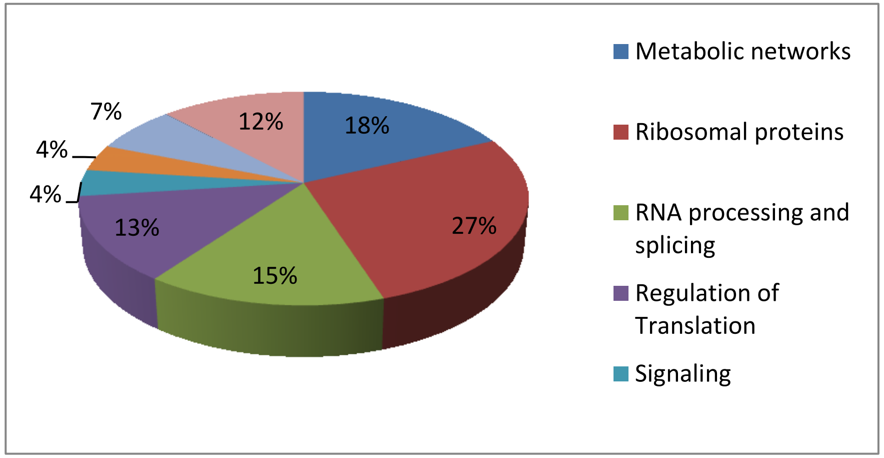

Supplement: Supplementary file 12 — Supplement_Table 4 [file 41419_2020_3251_MOESM12_ESM.pdf]
