# Supplementary material for: SEMG1/2 augment energy metabolism of tumor cells
Source: Cell Death Dis. 2020 Dec 11;11(12):1047. doi: 10.1038/s41419-020-03251-w (PMC7733513; doi:10.1038/s41419-020-03251-w)
Supplement: Supplementary file 13 — Supplement_Table 5 [file 41419_2020_3251_MOESM13_ESM.pdf]

PROTEINS ASSOCIATED ONLY WITH SEMG2						
			Number of peptides associated with corresponding recombinant protein			
**Identified protein**	**Accession Number**	**Molecular weight**	**GST**	**GST-SEMG1**	**GST-SEMG2**	
RPL29	P47914	18 kDa	0	0	**4**	60S ribosomal protein L29
ALYREF	Q86V81	27 kDa	0	0	**4**	THO complex subunit 4 OS
PSPC1	Q8WXF1	59 kDa	0	0	**5**	Paraspeckle component 1
