# Supplementary material for: SEMG1/2 augment energy metabolism of tumor cells
Source: Cell Death Dis. 2020 Dec 11;11(12):1047. doi: 10.1038/s41419-020-03251-w (PMC7733513; doi:10.1038/s41419-020-03251-w)
Supplement: Supplementary file 14 — Supplement_Table 6 [file 41419_2020_3251_MOESM14_ESM.pdf]

PROTEINS ASSOCIATED WITH BOTH SEMG1 and SEMG2							
			Number of peptides associated with corresponding recombinant protein				
Identified protein	Accession Number	Molecular weight	GST	GST-SEMG1	GST-SEMG2	Assigned number of protein function	
PCK2	Q16822	71 kDa	0	**7**	**3**	1	Phosphoenolpyruvate carboxykinase [GTP], mitochondrial
SHMT2	P34897	56 kDa	0	**8**	**3**	1	Serine hydroxymethyltransferase, mitochondrial
FASN	A0A0U1RQF0 (+1)	273 kDa	0	**17**	**4**	1	Fatty acid synthase
PKM	P14618	58 kDa	0	**23**	**14**	1	Pyruvate kinase PKM
RPL11	P62913	20 kDa	0	**3**	**3**	2	60S ribosomal protein L11
RPL35	P42766	15 kDa	0	**5**	**3**	2	60S ribosomal protein L35
RPS6	P62753	29 kDa	0	**5**	**4**	2	40S ribosomal protein S6
RPS17	P08708	16 kDa	0	**6**	**3**	2	40S ribosomal protein S17
RPL26	P61254 [3]	17 kDa	0	**8**	**6**	2	Cluster of 60S ribosomal protein L26
RPS4X	P62701	30 kDa	0	**9**	**3**	2	40S ribosomal protein S4, X isoform
RPS7	P62081	22 kDa	0	**10**	**4**	2	40S ribosomal protein S7
RPS5	P46782 (+1)	23 kDa	0	**10**	**8**	2	40S ribosomal protein S5
RPL23A	P62750	18 kDa	0	**11**	**6**	2	60S ribosomal protein L23a
DDX17	Q92841 (+1)	80 kDa	0	**3**	**2**	3	Probable ATP-dependent RNA helicase DDX17
NONO	Q15233	54 kDa	0	**3**	**11**	3	Non-POU domain-containing octamer-binding protein
PCBP1	Q15365	37 kDa	0	**5**	**4**	3	Poly(rC)-binding protein 1
RTCB	Q9Y3I0	55 kDa	0	**7**	**6**	3	tRNA-splicing ligase RtcB homolog
SFPQ	P23246	76 kDa	0	**7**	**14**	3	Splicing factor, proline- and glutamine-rich
SYNCRIP	O60506	70 kDa	0	**8**	**2**	3	Heterogeneous nuclear ribonucleoprotein Q
HNRNPA1	F8W6I7 (+1)	33 kDa	0	**8**	**4**	3	Heterogeneous nuclear ribonucleoprotein A1
PCBP1	Q15365 [5]	37 kDa	0	**9**	**4**	3	Cluster of Poly(rC)-binding protein 1
HNRNPA2B1	P22626	37 kDa	0	**9**	**8**	3	Heterogeneous nuclear ribonucleoproteins A2/B1
DDX5	P17844 (+1)	69 kDa	0	**9**	**9**	3	Probable ATP-dependent RNA helicase DDX5
DDX1	Q92499	82 kDa	0	**11**	**3**	3	ATP-dependent RNA helicase DDX1
HNRNPM	P52272	78 kDa	0	**13**	**5**	3	Heterogeneous nuclear ribonucleoprotein M
DDX3X	A0A0D9SF53 (+3)	81 kDa	0	**14**	**7**	3	ATP-dependent RNA helicase DDX3X
DDX17	Q92841 [4]	80 kDa	0	**15**	**13**	3	Probable ATP-dependent RNA helicase DDX17

HNRNPU	Q00839	91 kDa	0	**20**	**5**	3	Heterogeneous nuclear ribonucleoprotein U
HNRNPM	P52272	78 kDa	0	**23**	**15**	3	Heterogeneous nuclear ribonucleoprotein M
PDIA3	P30101	57 kDa	0	**4**	**8**	4	Protein disulfide-isomerase A3
CSDE1	O75534	89 kDa	0	**7**	**3**	4	Cold shock domain-containing protein E1
HIST1H1T	P22492 [2]	22 kDa	0	**3**	**4**	6	Cluster of Histone H1t
CRIP2	H0YFA4 (+1)	21 kDa	0	**3**	**4**	6	Cysteine-rich protein 2
LRRC59	Q96AG4	35 kDa	0	**10**	**5**	6	Leucine-rich repeat-containing protein 59
NCL	P19338	77 kDa	0	**5**	**4**	6	Nucleolin
EEF2	P13639	95 kDa	0	**18**	**9**	6	Elongation factor 2
MYO6	A0A0A0MRM8	145 kDa	0	**5**	**3**	6	Unconventional myosin-VI
USP10	Q14694	87 kDa	0	**3**	**3**	6	Ubiquitin carboxyl-terminal hydrolase 10

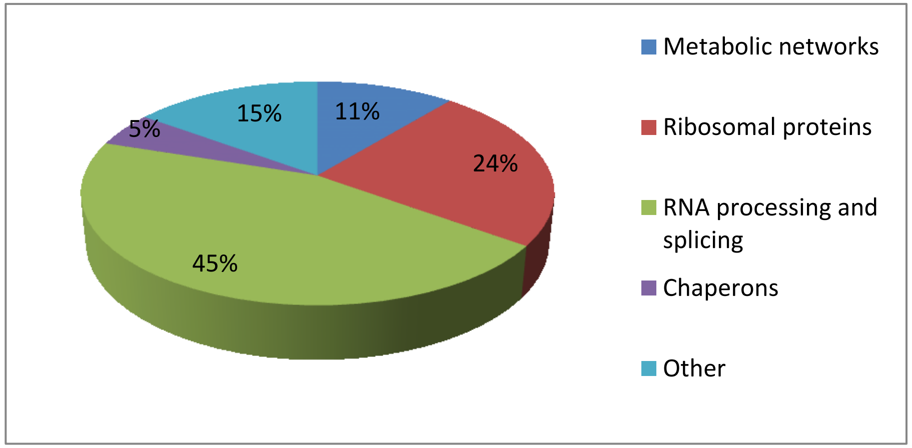

Assigned number of protein function	Protein function	Number of proteins identified	Percentage
1	Metabolic networks	4	**11**
2	Ribosomal proteins	9	**24**
3	RNA processing and splicing	16	**45**
4	Chaperons	2	**5**
	Other	7	**15**
